# Combination of anlotinib with immunotherapy enhanced both anti-angiogenesis and immune response in high-grade serous ovarian cancer

**DOI:** 10.3389/fimmu.2025.1539616

**Published:** 2025-04-07

**Authors:** Hongwei Lan, Hui Liu, Helei Hou, Chuantao Zhang, Jingjuan Zhu, Na Zhou, Xiaochun Zhang

**Affiliations:** ^1^ Precision Medicine Center of Oncology, The Affiliated Hospital of Qingdao University, Qingdao, Shandong, China; ^2^ Department of Clinical Laboratory, Qingdao Women’s and Children’s Hospital, Qingdao, Shandong, China; ^3^ Department of Oncology, The Affiliated Hospital of Qingdao University, Qingdao, Shandong, China

**Keywords:** high-grade serous ovarian cancer, patient-derived xenograft model, anlotinib, immunotherapy, single-cell RNA sequencing

## Abstract

**Background:**

High-grade serous ovarian cancer (HGSOC) poses significant treatment challenges due to frequent recurrence and resistance to conventional therapies. Combination of anlotinib with immunotherapy have showed promise in various cancers, but its impact on HGSOC remains to be fully elucidated.

**Methods:**

A retrospective analysis was performed on 36 HGSOC patients treated with anlotinib-based therapies, including both monotherapy and combination treatment with anti-PD-L1/anti-PD-1 antibody (aPD-L1/aPD-1). Peripheral blood mononuclear cell-derived patient-derived xenograft (PBMC-PDX) model was established from drug-resistant recurrent HGSOC patient-derived tumor cells, and single-cell RNA sequencing (scRNA-seq) was conducted to dissect the TME following treatment with anlotinib, anlotinib + aPD-L1 and anlotinib + aPD-1.

**Results:**

Clinical analysis revealed a disease control rate (DCR) of 71.43% for anlotinib monotherapy, which improved to 100% when combined with aPD-L1/aPD-1. In PBMC-PDX models, treatment evaluation showed that anlotinib decreased tumor volume, an effect further enhanced by its combination with aPD-L1. scRNA-seq analysis demonstrated that anlotinib reduced the proportions of myofibroblastic cancer-associated fibroblasts and ESM1^+^ endothelial cells, resulting in decreased angiogenesis. The combination of anlotinib and aPD-L1 further amplified these effects, promoting CD8^+^ T cell infiltration and reversing T cell exhaustion, whereas anlotinib + aPD-1 showed limited efficacy in this regard. Additionally, anlotinib + immunotherapy induced a shift toward M1 polarization of myeloid cells, enhanced anti-tumor activity, and inhibited immune escape. Cell-cell communication analysis revealed reduced APP-CD74 signaling and increased CD99-CD99 signaling, which might contribute to immune activation.

**Conclusion:**

The combination of anlotinib and aPD-L1 effectively modulates the HGSOC tumor microenvironment by inhibiting angiogenesis, enhancing immune infiltration, and reversing T cell exhaustion.

## 1 Introduction

Ovarian cancer remains a significant global health challenge, with approximately 314,000 new cases diagnosed and 207,000 deaths annually—of which high-grade serous ovarian cancer (HGSOC) accounts for over 80% (1, 2). Despite decades of research and therapeutic advancements, the cornerstone treatment for HGSOC—surgical intervention followed by platinum-based chemotherapy—faces substantial hurdles, including high recurrence rates and the eventual development of resistance. Consequently, the 5-year survival rate remains below 40% (3, 4). This therapeutic impasse has intensified the search for novel strategies, such as targeted therapies and immunotherapies, to overcome the limitations of current standard care.

Anlotinib is a novel oral multi-targeted tyrosine kinase inhibitor (TKI) that exerts antitumor effects primarily by inhibiting angiogenesis and targeting pathways involved in tumor growth and survival (5). Initially approved for the treatment of recurrent, locally advanced, or metastatic non-small cell lung cancer (NSCLC) (6), anlotinib has demonstrated promising efficacy in clinical trials for medullary thyroid carcinoma and soft tissue sarcoma (7, 8). Emerging studies have reported that anlotinib exhibits favorable antitumor activity and acceptable safety profiles in ovarian cancer (9, 10), with several clinical trials currently underway. Notably, combinations of anlotinib with other therapies—including chemotherapy, immunotherapy, and radiotherapy—have shown enhanced anticancer effects compared to monotherapy (11, 12). However, limited research on the roles of anlotinib and anti-PD-L1/anti-PD-1 antibody (aPD-L1/aPD-1) within the ovarian cancer tumor microenvironment (TME) has left their clinical efficacy and underlying mechanisms unclear, hindering broader clinical application and regulatory approval.

This study comprehensively evaluates the therapeutic effects of anlotinib in drug-resistant recurrent HGSOC, both as a monotherapy and in combination with aPD-L1/aPD-1. By analyzing real-world clinical treatment data, constructing an HGSOC Peripheral blood mononuclear cell-derived patient-derived xenograft (PBMC-PDX) model, and performing single-cell sequencing (scRNA-seq), this research assesses the changes in the HGSOC TME and the underlying mechanisms, aiming to provide a deeper understanding and more effective treatment strategies for drug-resistant HGSOC.

## 2 Methods

### 2.1 Patient selection and evaluation criteria

We conducted a retrospective study of patients with HGSOC who received anlotinib-based therapy at our institution between January 2020 and July 2024. Inclusion criteria required a confirmed diagnosis of HGSOC, an Eastern Cooperative Oncology Group (ECOG) performance status of 0–2, completion of at least three cycles of anlotinib treatment, and availability of comprehensive medical records. Clinical data were collected through medical chart reviews and patient follow-ups. All treatments were administered in accordance with relevant clinical guidelines and drug protocols. PD-L1 expression levels were assessed by immunohistochemistry using the PD-L1 22C3 antibody. PD-L1 positivity was defined as a tumor proportion score (TPS) or a combined positive score (CPS) greater than 1%. Tumor staging prior to treatment was determined according to the guidelines of the 8th edition of the American Joint Committee on Cancer (AJCC). Treatment responses were evaluated radiologically using the Response Evaluation Criteria in Solid Tumors (RECIST) version 1.1.

### 2.2 Obtaining and processing of HGSOC sample

With approval from the Ethics Committee of Qingdao University Affiliated Hospital and after obtaining informed consent, we collected a 700 mL sample of malignant ascites from a patient diagnosed with advanced HGSC. The cancer had metastasized to the liver, peritoneum, pelvis, and multiple lymph nodes. The patient had previously undergone tumor debulking surgery, followed by multiple times of adjuvant chemotherapy. Despite these interventions, the disease progressed, evidenced by increasing tumor size and ascites, leading to a diagnosis of progressive disease (PD). Genetic profiling revealed a TP53 mutation, MYC amplification, a low tumor mutational burden of 7.1 mut/Mb, microsatellite stability, and low PD-L1 expression (TPS < 1%). No mutations in BRCA1 or BRCA2 were detected. Written informed consent was obtained from the patient for the publication of any potentially identifiable images or data included in this article.

### 2.3 Construction of PBMC-PDX models

To generate the PDX model, ascitic fluid was processed in a biosafety cabinet. The fluid was centrifuged at 500 × g for 5 minutes to pellet the cells. After discarding the supernatant, red blood cell lysis buffer was added, followed by a 5-minute incubation at room temperature. The remaining cells were washed twice with phosphate-buffered saline (PBS) and centrifuged. The enriched tumor cells were then resuspended in pre-chilled PBS. A 1:1 mixture of Matrigel and tumor cell suspension was prepared, and the cells were subsequently injected subcutaneously into NOD/SCID/IL2Rγ(NSG) mice. Tumor growth was monitored at regular intervals, with volumes calculated using the formula:


$$V=\frac{Length\times Width^{2}}{2}$$

Upon reaching an appropriate size, a portion of the tumor was excised for pathological assessment to ensure consistency with the original tumor type. The established PDX model was then maintained in NSG mice for further experiment. To mimic a fully intact immune microenvironment, PBMCs (Miaoshun Biotechnology Co., Shanghai, China) were administered intravenously at a dose of 0.2 mL containing 5 × 10^6^ cells per mouse. Flow cytometry (FACS) analysis confirmed the successful establishment of the PBMC-PDX model, which was then used for subsequent drug efficacy assessments.

### 2.4 Drugs

Anlotinib was purchased from Chia Tai Tianqing Pharmaceutical Group Co. (Lianyungang, China). The PD-L1-specific antibody atezolizumab (1200 mg/20 mL) was purchased from Roche (Basel, Switzerland). The PD-1-specific antibody toripalimab (240 mg/5 mL) was purchased from Suzhou Zhenhe Biomedical Pharmaceutical Co. (Suzhou, China).

### 2.5 Animal study

Female NSG mice (6–8 weeks old, 18–20 g) were obtained from Shanghai Model Organisms Center, Inc. (Shanghai, China). Mice were housed in a specific pathogen-free environment in individually ventilated cages under controlled conditions of temperature (20–26 °C), humidity (40–70%), and a 12-hour light/dark cycle. Three mice were housed per cage (dimensions: 325 mm × 210 mm × 180 mm) with sterilized corn cob bedding that was replaced twice weekly. Mice had ad libitum access to food and water; the feed was sterilized by Co-60 irradiation, and the water was sterilized under high pressure, both replenished twice weekly. Tumor-bearing mice were euthanized when they exhibited severe deterioration in condition or when tumors reached an average volume of 2,000 mm³. At the conclusion of the study, all mice were euthanized. All experimental procedures adhered to the guidelines of the Institutional Animal Care and Use Committee (IACUC) and received prior approval.

### 2.6 Fluorescence-activated cell sorting

Fluorescence-Activated Cell Sorting (FACS) analysis was carried out using an Attune^®^ NxT Acoustic Focusing Cytometer (Life Technologies, CA, USA). The antibodies employed included: APC-conjugated anti-human CD45, APC/Cy7-conjugated anti-human CD8, PE-conjugated anti-human CD3, PE-conjugated anti-human CD56 from Biolegend (CA, USA), and BV421-conjugated anti-human CD45, BB515-conjugated anti-human CD3, and BV605-conjugated anti-human CD4 from BD Biosciences (NJ, USA).

Blood samples from treated PBMC-PDX model mice were divided into seven tubes (100 μL each), processed as follows: 1) Blank control, 2) CD45 single stain (5 μL anti-human CD45 antibody), 3) CD3 single stain (5 μL anti-human CD3 antibody), 4) CD4 single stain (5 μL anti-human CD4 antibody), 5) CD8 single stain (5 μL anti-human CD8 antibody), 6) CD56 single stain (5 μL anti-human CD56 antibody), and 7) a mixture of 5 μL each of CD45, CD3, CD4, CD8, and CD56 antibodies. The gating strategy used was R1: total cells, R2: single cells, R3: CD45+ cells, R4: CD45+CD3+ cells, R5: CD4+ cells, R6: CD8+ cells, and R7: CD45+CD56+ cells.

### 2.7 Single-cell RNA sequencing and analysis

Tumor tissues were cut into 1-2 mm³ fragments and digested using the SoloTM Tumor Dissociation Kit (Sinotech Genomics, JZ-SC-58201) for 60 minutes at 37°C. The resulting single-cell suspension was filtered through a 40 μm strainer and kept on ice until further single-cell transcriptome analysis. The digestion was halted with RPMI-1640.

The single-cell transcriptome analysis followed the protocol of the BD Rhapsody system (BD Biosciences, CA). The cells were first stained with calcein AM and Draq7 for accurate determination of cell concentration and viability using the BD Rhapsody™ Scanner. They were then loaded into a microwell cartridge, followed by an excess loading of cell capture beads. After cell lysis with lysis buffer, the beads were retrieved and washed to prepare for reverse transcription.

Using the BD Rhapsody cDNA Kit (BD Biosciences, Cat. No. 633773) and BD RhapsodyTM WTA Amplification Kit (BD Biosciences, Cat. No. 633801), a cDNA library with cell labels and UMI information was created based on the microbead-captured single-cell transcriptome. Sequencing was carried out in PE150 mode (paired-end 150 bp reads) on the NovaSeq platform. The raw sequencing data were processed through the BD Rhapsody Whole Transcriptome Assay Analysis Pipeline (v1.8), which includes quality filtering, read and molecule annotation, and putative cell identification. The GRCh38 genome was used as the reference for this pipeline.

For subsequent clustering analysis and visualization, R software (v4.3.0) (13) and the Seurat R package (v5.0.3) (14) were employed. Cells with over 25% mitochondrial UMI or fewer than 500 UMI or 200 genes were excluded. The gene expression matrix was normalized according to the total cellular UMI count, and 2000 highly variable features were selected for PCA after data scaling based on UMI counts. The first 50 principal components were then used for clustering at a resolution of 0.6, utilizing t-SNE or UMAP algorithms. To visualize gene expression in each cluster, feature plots, violin plots, and heatmaps were generated.

Cluster-specific markers were identified through the FindAllMarkers function using the Wilcoxon test, with a threshold of log2-fold change > 0.25 and min. pct > 0.25. Each cluster was annotated with canonical marker genes from prior literature to unbiasedly identify the cell types in the filtered and combined datasets. Gene Ontology (GO) functional enrichment was performed using the ClusterProfiler R package (v4.4.4) (15), while Hallmark and KEGG pathway enrichment analyses were conducted using the GSVA (v1.50.5) (16) and msigdbr (v7.5.1) (17) R packages. SCENIC (v1.3.1) (18) was employed for transcription factor analysis, and CellChat (v1.6.1) (19) was utilized for cell-cell communication analysis. Pseudotime trajectory analysis was carried out using the Monocle2 R package (v2.18.0) (20) and CytoTRACE (v0.3.3) (21). The CHPF algorithm was applied to identify hypoxic states in cells (22).

### 2.8 Spatial transcriptome analysis and prognosis analysis

Spatial transcriptome data for HGSOC were obtained from the GSE211956 dataset (23). Quality control and subsequent analyses were performed using standard protocols provided by the Seurat package. Kaplan-Meier analysis of NR3C1 expression and patient prognosis after aPD-L1/aPD-1 treatment, were conducted using data from the Kaplan-Meier Plotter database (24).

### 2.9 Immunohistochemistry and immunofluorescence

Tumor tissues were fixed in 4% paraformaldehyde, dehydrated in graded ethanol, and embedded in paraffin. Sections (5 μm) were deparaffinized, rehydrated, and rinsed with PBS. For immunohistochemistry, antigen retrieval was performed using Tris-EDTA buffer (pH 9.0) at 95°C for 10 minutes. Sections were incubated with rabbit anti-human CD8 alpha antibody (1:1000, Abcam) or anti-human PD-L1 antibody (1:800, Invitrogen), followed by detection with the HRP/DAB IHC Detection Kit (Abcam) according to the manufacturer’s instructions. Rabbit IgG isotype control (Cell Signaling Technology) was used as a negative control.

For immunofluorescence, tumor sections were incubated with anti-CD31 antibody (1:500, Servicebio Technology), followed by incubation with fluorophore-conjugated secondary antibodies (1:1000, Servicebio Technology).

### 2.10 Statistical analysis

Data are presented as the mean ± SEM. Statistical analyses of gene expression or module scores among groups used either the Mann–Whitney test or an unpaired two-tailed Student’s t-test, with p-values reported where relevant. Multiple-testing correction for differentially expressed genes was implemented by the corresponding R packages. Genes with adjusted p-values< 0.05 were considered significant. Correlation analyses employed the R function cor.test with Pearson’s method. Differential expression in pseudotime or cell-type trajectories used negative binomial models with q-values< 0.01 indicating significance. GSVA was performed with the ssgsea method on normalized gene expression data. Most of code and statistical computations were done primarily in R, with some steps handled in Python and GraphPad Prism. Statistical significance was defined as a *P*< 0.05.

## 3 Results

### 3.1 Clinical characteristics and treatment response of patients receiving anlotinib-based treatments

Based on the inclusion and exclusion criteria, 36 refractory HGSOC patients who received anlotinib treatment were selected (**Supplementary Figure S1**). The median age was 66 years, with the majority presenting at advanced stages: 55.56% were at stage IV and 30.56% at stage III. Among these patients, only 10 underwent PD-L1 testing, of whom 70% were PD-L1 positive (representing 19.44% of the total cohort). P53 mutation testing was performed on 24 patients, revealing that 75% (18 out of 24) harbored P53 mutations. These findings align with prior studies, indicating a high prevalence of PD-L1 positivity and P53 mutations in HGSOC (25, 26). Patients received different anlotinib regimens: 38.89% underwent monotherapy, and 16.67% received anlotinib combined with immunotherapy. Notably, anlotinib was predominantly used in patients who had relapsed or were refractory to platinum-based therapy, with 50% initiating anlotinib at the fourth line of treatment or beyond. The most common side effects were rash and fatigue, both managed symptomatically without treatment interruption. Only one patient discontinued anlotinib due to gastrointestinal bleeding (**Table 1**).

**Table 1:** Demographic and baseline characteristics of the HGSOC patients received anlotinib-based treatments.

Characteristics	Value(N=36)
Median age(range)-year	66 (44-87)
Stage-no. (%)	
I	1 (2.78%)
II	4 (11.11%)
III	11 (30.56%)
IV	20 (55.56%)
PD-L1 status-no. (%)	
Positive	7 (19.44%)
Negative	3 (8.33%)
Not available	26 (72.22%)
P53 status-no. (%)	
Mutation	18 (50.00%)
Wild	6 (16.67%)
Not available	12 (33.33%)
Anlotinib therapy strategy-no. (%)	
Mono-therapy	14 (38.89%)
+ Targeted therapy	
Olaparib	7 (19.44%)
Niraparib	4 (11.11%)
+ Immunotherapy	
Sintilimab	3 (8.33%)
Pembrolizumab	1 (2.78%)
Atezolizumab	1 (2.78%)
Durvalumab	1 (2.78%)
+ Chemotherapy	3 (8.33%)
+ Chemotherapy & Targeted therapy (Niraparib)	2 (5.56%)
The line of anlotinib therapy -no. (%)	
2	7 (19.44%)
3	11 (30.56%)
4	8 (22.22%)
5	6 (16.67%)
≥6	4 (11.11%)
Adverse effect -no. (%)	
Rash (G1,2; G2,1)	3 (8.33%)
Fatigue	3 (8.33%)
Hemorrhage	1 (2.78%)
Congestion	1 (2.78%)
Hypertension	1 (2.78%)

**Figure 1:**
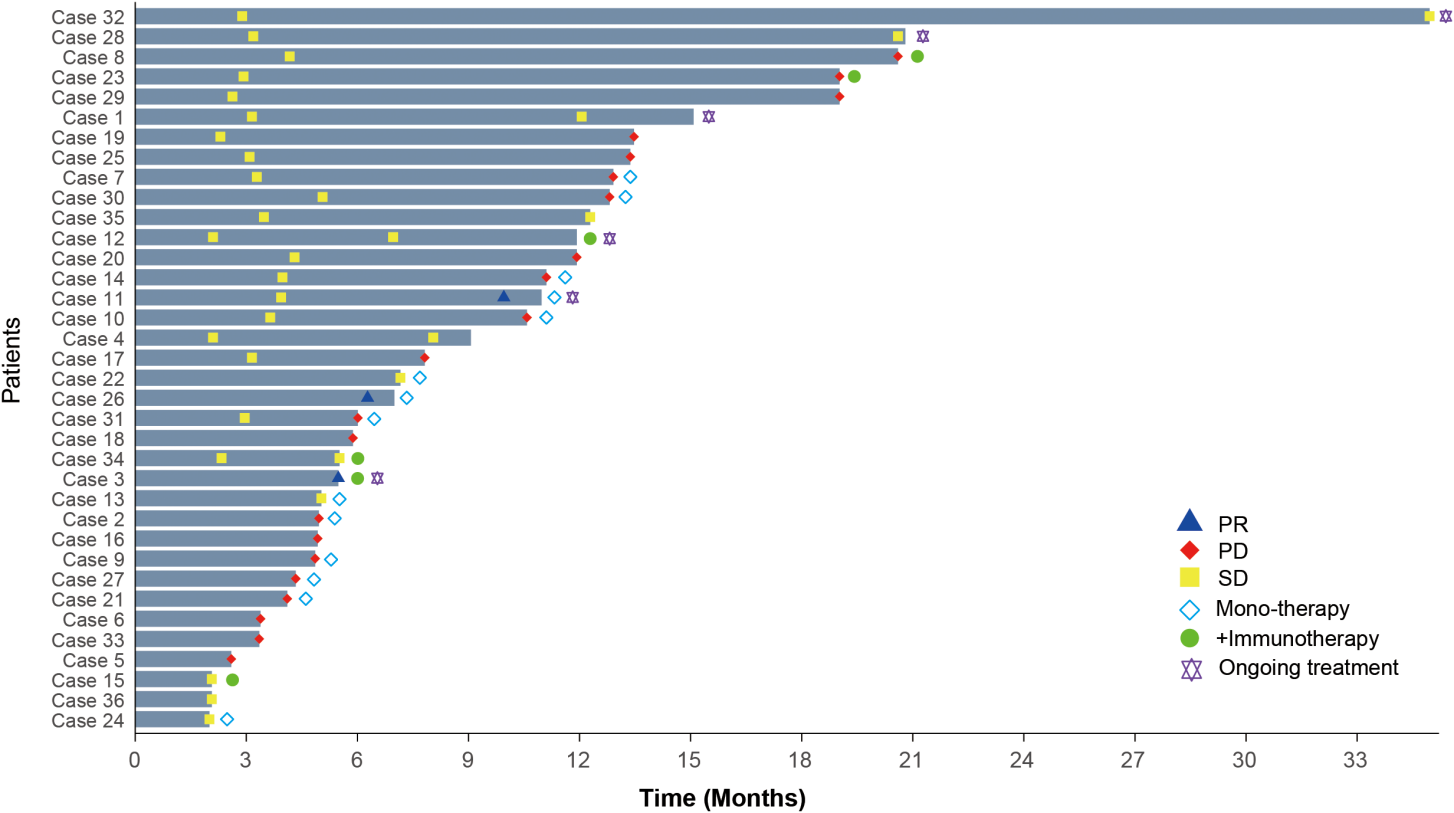
Treatment duration and clinical responses in HGSOC patients receiving anlotinib-based therapies. PR, Partial response; PD, Progressive disease; SD, Stable disease.

The overall disease control rate (DCR) was 75% (95% CI: 58.93–86.25), and the objective response rate (ORR) was 8.33% (95% CI: 2.87–21.83). In the monotherapy group, the DCR was 71.43% (95% CI: 45.35–88.28) with an ORR of 14.29% (95% CI: 4.01–39.94). In contrast, the combination immunotherapy group achieved a DCR of 100% (95% CI: 60.97–100) and an ORR of 16.67% (95% CI: 3.01–56.35). At the time of the final follow-up, the median progression-free survival (PFS) was 7.5 months (range: 2–34.9 months), while the median overall survival (OS) had not yet been reached. The monotherapy group had a median PFS of 6.5 months (range: 2–12.91 months), compared to 8.7 months (range: 2.1–20.6 months) in the combination immunotherapy group (**Figure 1**). These findings are consistent with those reported for anlotinib and its combination with immune checkpoint inhibitors (ICIs) in advanced NSCLC, where combination therapy achieved higher DCRs and longer PFS compared to monotherapy (27, 28).

These findings suggest that anlotinib, particularly in combination with immunotherapy, holds therapeutic potential for recurrent or refractory HGSOC. However, given the lack of approved indications for anlotinib or ICIs in HGSOC, clinical trials remain challenging. Thus, constructing PDX model was the most feasible approach to further evaluate this combination therapy in a preclinical setting.

### 3.2 Establishment and treatment of the immunocompetent patient-derived xenograft model

**Figure 2:**
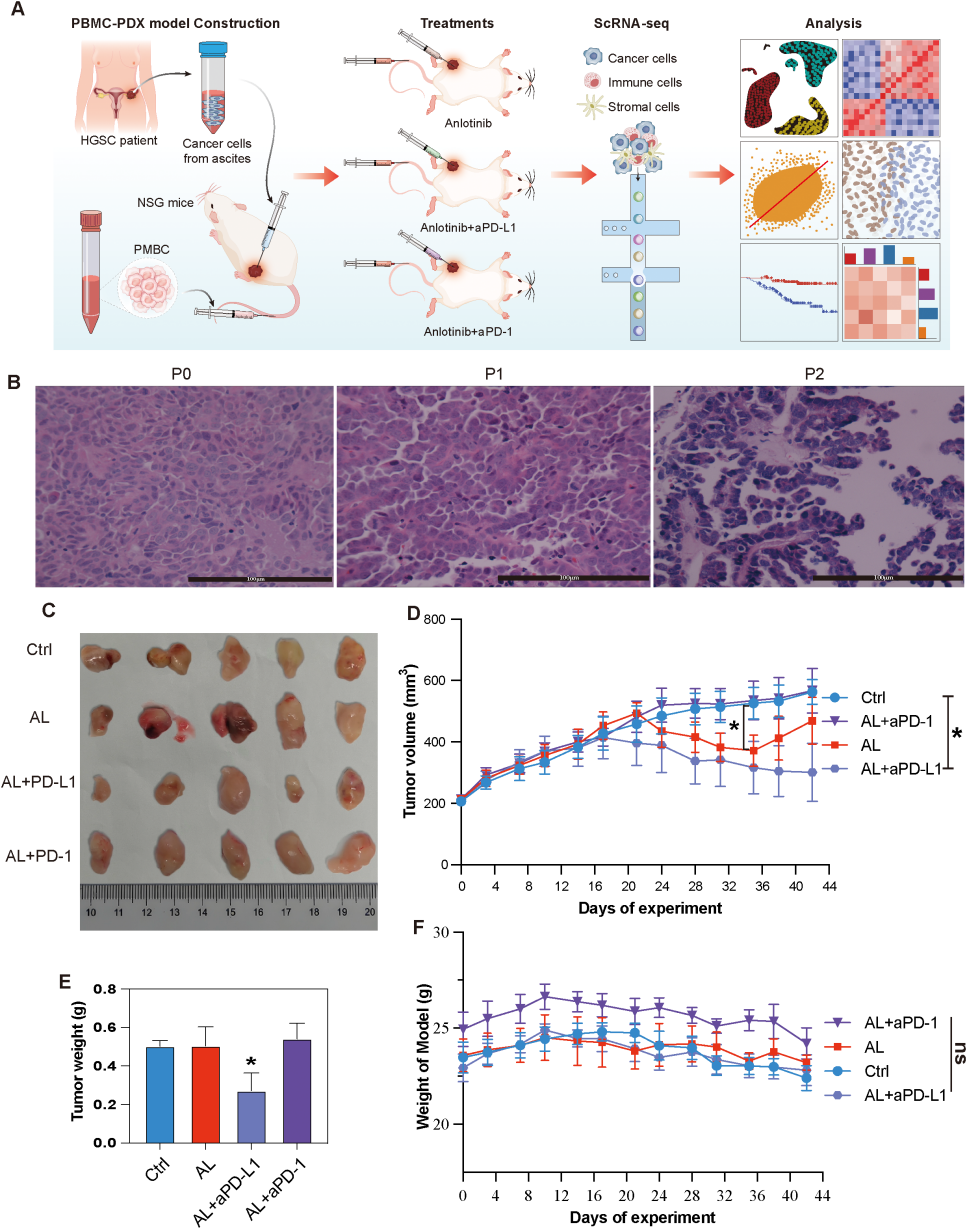
Establishment and treatment of HGSOC PBMC-PDX models. **(A)** Schematic representation of the study design and analytical workflow. **(B)** HE staining of the patient tumor (primary tumor) and the first and second passages of the PDX model. **(C)** Representative tumor tissue samples from different PBMC-PDX groups. **(D)** Changes in tumor volume over time among different groups. **(E)** Comparison of tumor weights among different groups at the end of the study. **(F)** Body weight variations of PBMC-PDX model mice among different groups. * indicates P< 0.05, ** indicates P< 0.01, *** indicates P< 0.001, and ns means no significant (applied to all subsequent figures).

PDX models largely retain the characteristics of the parental tumors and exhibit high similarity between samples, making them more suitable for drug testing than traditional cell lines (29). To establish the PDX model, tumor cells were isolated from the malignant ascites of a refractory HGSOC patient who had relapsed after multiple lines of platinum-based chemotherapy. These cells were processed and subcutaneously injected into immunodeficient NSG mice (Methods) (**Figure 2A**). Pathological analysis confirmed that the transplanted tumor was HGSOC and closely resembled the parent tumor, with serial transplantation demonstrating stable passaging capability (**Figure 2B**).

To construct an immunocompetent PDX model, human PBMCs were subsequently injected into the PDX models (Methods). Twenty models were randomly and equally divided into four groups. The experimental groups received either anlotinib monotherapy, anlotinib combined with aPD-L1or aPD-1 (**Supplementary Table S1**). The results indicated that anlotinib monotherapy initially decreased tumor volume but showed progression during the final week of the experiment. In contrast, the combination of anlotinib with aPD-L1 resulted in a more pronounced and sustained reduction in tumor volume, along with a significant decrease in tumor weight. However, the combination of anlotinib with aPD-1 exhibited a less apparent reduction in tumor volume (**Figures 2C–E**). Despite the random grouping resulting in the anlotinib + aPD-1 group having a slightly higher initial body weight than the other groups, none of these treatments led to significant changes in the body weight of the models (**Figure 2F**).

Given the observed differences between the effects of AL + aPD-1 and AL + aPD-L1, we sought to uncover the cellular and molecular mechanisms underlying these discrepancies. scRNA-seq was employed to comprehensively analyze changes in the TME and evaluate the mechanisms by which this combination therapy exerts its antitumor effects in HGSOC.

### 3.3 Single-Cell RNA sequencing reveals cellular composition of HGSOC tumor microenvironment

**Figure 3:**
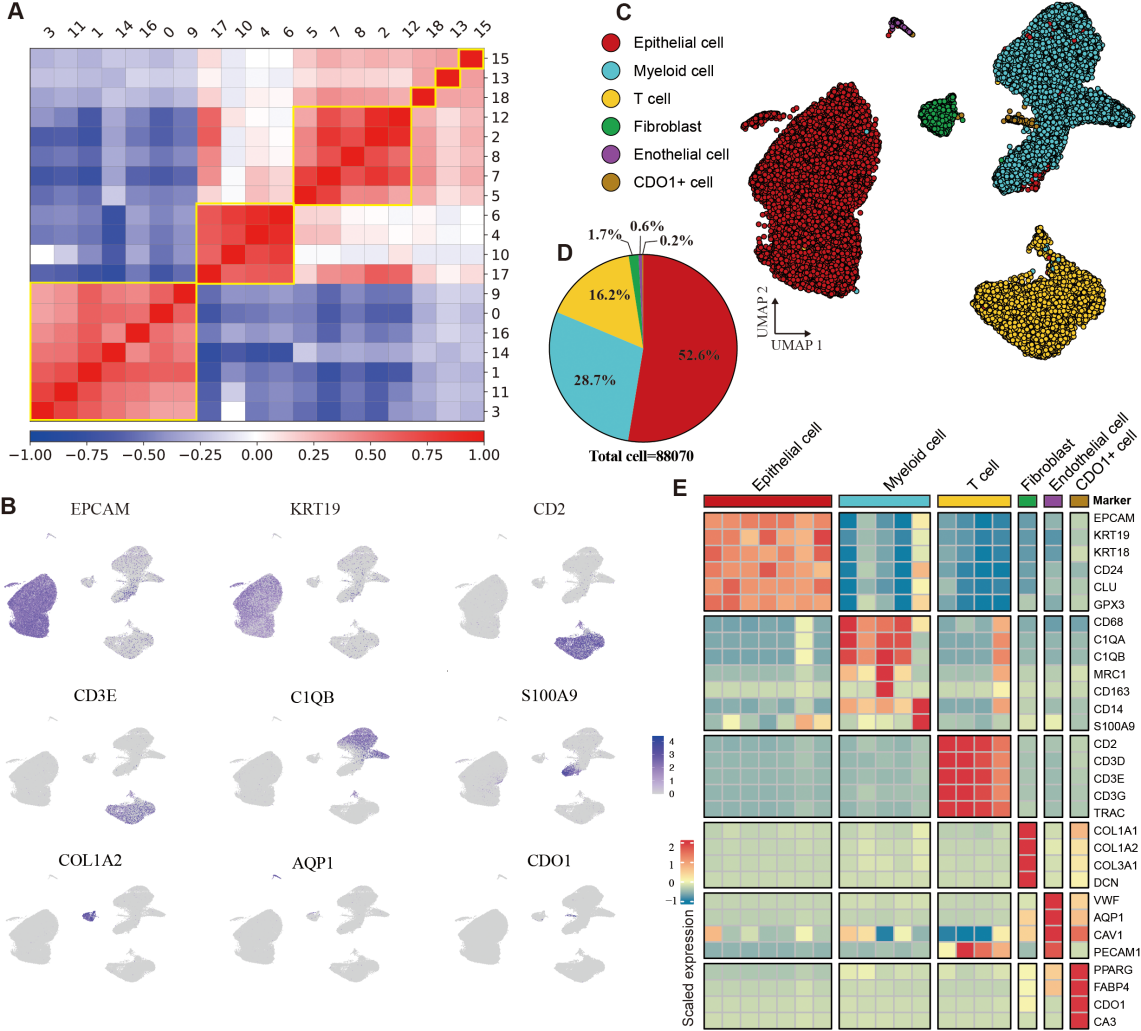
Identification of cell types in the HGSOC tumor microenvironment. **(A)** Heatmap depicting the Pearson correlation coefficients among identified cell clusters. **(B)** Uniform Manifold Approximation and Projection (UMAP) visualization of cells colored by the expression levels of marker genes. **(C)** UMAP plot illustrating the main cell types identified in the tumor microenvironment (TME). **(D)** Pie chart displaying the proportion of different cell types within the dataset. **(E)** Heatmap showing gene expression profiles across six cell types. See also **Supplementary Figure S2**.

Eight tumor samples were collected and processed for scRNA-seq using standard protocols (Methods). After quality control, batch effect correction, and dimensionality reduction clustering (**Supplementary Figures S2A, B**), we identified a total of 19 distinct cell clusters (**Supplementary Figure S2C**). Through pearson correlation analysis and marker gene expression profiling, these clusters were categorized into epithelial cells, myeloid cells, T cells, fibroblasts, endothelial cells, and CDO1^+^ cells (**Figures 3A–C**). Epithelial cells constituted the largest proportion, accounting for 52.6% of the total cells, followed by myeloid cells at 28.7% and T cells at 16.2%. The stromal cell population was primarily composed of fibroblasts and endothelial cells (**Figure 3D**, **Supplementary Figure S2D**). The epithelial cells exhibited high expression of EPCAM, KRT19, and KRT18; myeloid cells were marked by elevated expression of CD68, C1QA, and S100A9. T cells were characterized by high levels of CD2, CD3D, and CD3E, while fibroblasts showed strong expression of COL1A1 and COL1A2. Endothelial cells were identified by high expression of VWF and PECAM1. Additionally, CDO1^+^ cells displayed elevated expression of CDO1, PPARG, and FABP4 (**Figure 3E**). Additionally, IHC results confirmed the absence of B cells and NK cells in the model (**Supplementary Figure S2E**).

### 3.4 Combination of anlotinib and immunotherapy alters tumor cell heterogeneity

**Figure 4:**
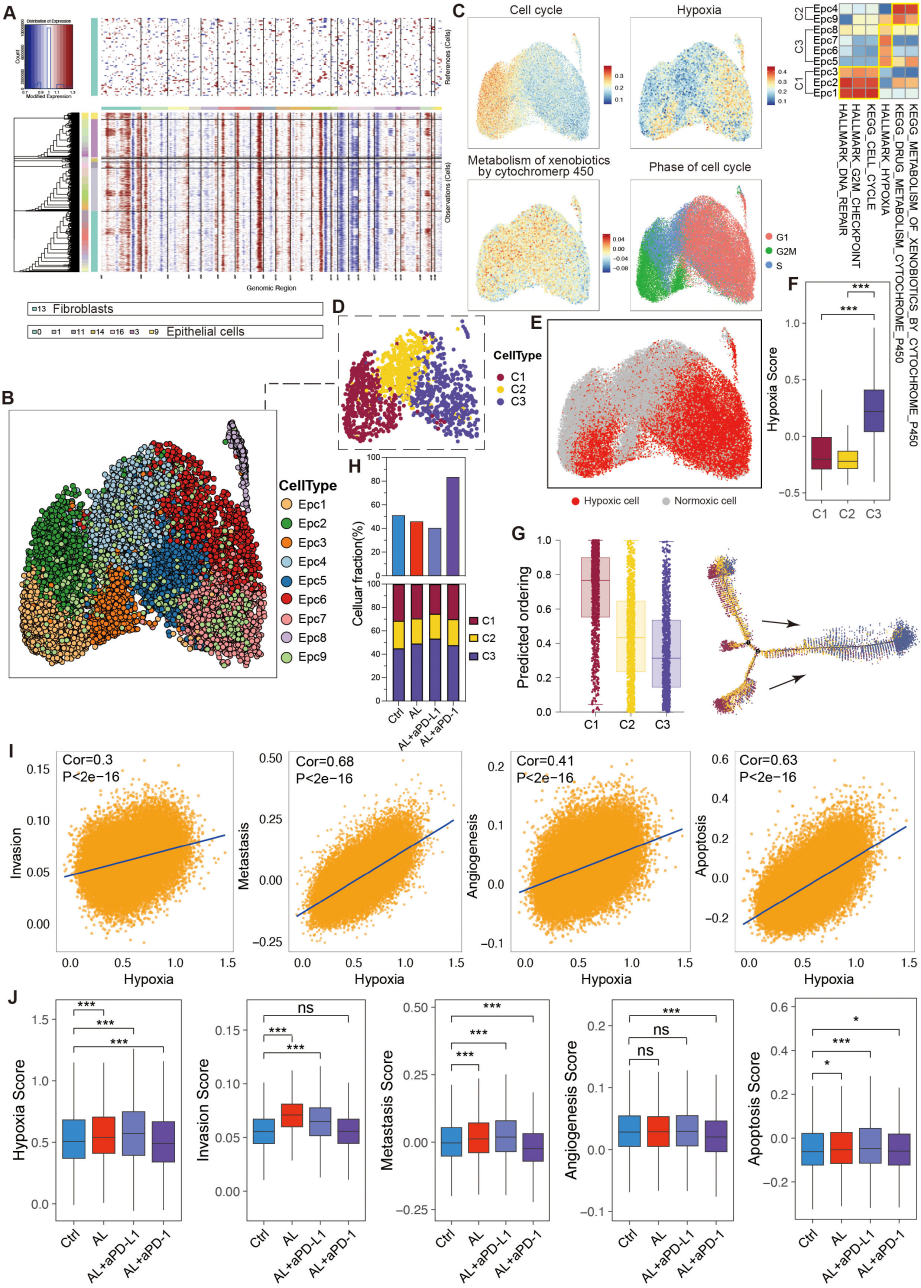
Heterogeneity and variation of epithelial cells in the HGSOC TME. **(A)** Hierarchical heatmap from InferCNV analysis displaying large-scale copy number variations (CNVs) in epithelial cells. **(B)** UMAP visualization illustrating the clustering of epithelial cell subtypes. **(C)** UMAP and corresponding heatmap depicting enriched signaling pathways across epithelial cell subtypes. **(D)** Classification of epithelial cell subtypes into three main cell types based on their biological characteristics. **(E, F)** Confirmation of the hypoxic status of epithelial cells using the CPHF algorithm. **(G)** Pseudotime trajectory analysis of epithelial cell subtypes. **(H)** Proportions of epithelial cells and their sub-types across different groups. **(I)** Analysis of the relationship between tumor cell hypoxia and invasion, metastasis, angiogenesis, and apoptosis. **(J)** Comparison of hypoxia, invasion, metastasis, angiogenesis, and apoptosis score in tumor cells following different treatments. See also **Supplementary Figure S3**.

To further explore tumor cell heterogeneity, we conducted an in-depth analysis of the epithelial cell population. Using inferCNV analysis, we discovered that nearly all epithelial cells exhibited malignancy (**Figure 4A**), which may be facilitated by the comprehensive extraction of tumor tissues from the PBMC-PDX model. Dimensionality reduction and clustering further divided these tumor cells into nine subtypes (Epc1–Epc9) (**Figure 4B**), each with distinct gene expression profile (**Supplementary Figure S3A**). Based on biological characteristics, these epithelial cells were categorized into three main types: C1 cells, primarily consisting of proliferative groups (Epc1, Epc2, Epc3); C2 cells, associated with drug metabolism (Epc4, Epc9); and C3 cells, mainly related to hypoxia (Epc5, Epc6, Epc7, Epc8) (**Figure 4C**). Utilizing seven hypoxia-related genes identified through the CHPF algorithm, we further classified the cells into hypoxic and non-hypoxic types, confirming the hypoxic phenotype of C3 (**Figure 4D–F**).

Pseudotime analysis indicated that C1 cells possessed higher differentiation potential, allowing them to proliferate and develop into C2 and C3 cells, whereas C3 cells exhibited the lowest differentiation potential. Genes such as CAV2 played crucial roles in this developmental trajectory (**Figure 4G**, **S3B**). The biological differences among C1, C2, and C3 cells were governed by specific transcription factor regulons. Specifically, the E2F family of proliferative transcription factors, known to play a key role in cell cycle progression and proliferation, displayed markedly higher activity and expression in C1 cells, while ATF4 and ATF6, transcription factors related to hypoxia, were significantly upregulated in C3 cells (**Supplementary Figures S3C, D**).

After treatment with anlotinib, there was a reduction in the tumor cell proportion, and the combination of anlotinib with aPD-L1 further decreased this proportion. Conversely, the combination of anlotinib with aPD-1 led to an increase in tumor cell proportion (**Figure 4H**), corroborating previous animal experiments that demonstrated the therapeutic efficacy of anlotinib in HGSOC and the enhanced effect when combined with aPD-L1, while anlotinib + aPD-1 showed no significant antitumor activity.

Additionally, the various treatment regimens differentially influenced the composition of tumor cells. Specifically, anlotinib significantly reduced the proportion of C1 cells while increasing the proportion of C3 cells, with the combination of aPD-L1 amplifying this effect, suggesting that anlotinib+aPD-L1 exhibited enhanced effects in inhibiting tumor cell proliferation and promoting anti-angiogenesis. Notably, although the combination of anlotinib with aPD-1 did not yield significant therapeutic benefits, it did result in a reduction in the C1 cell proportion and an increase in C3 cells to a certain degree (**Figure 4H**).

Anlotinib treatment also led to a reduction in the expression of the E2F transcription factor family, suggesting that its antitumor effects may be mediated through both the suppression of cell proliferation and anti-angiogenesis. Surprisingly, the combination of anlotinib with aPD-L1 markedly increased the expression of ATF4 and ATF6 (**Supplementary Figure S3E**), suggesting its enhanced anti-angiogenesis capability.

Further analysis revealed that hypoxia can drive tumor cells to acquire enhanced invasive, metastatic, and angiogenic capabilities while simultaneously promoting tumor cell apoptosis (**Figure 4I**). Following treatment with anlotinib or anlotinib + aPD-L1, tumor cells exhibited increased hypoxia and apoptosis, accompanied by enhanced invasive potential (**Figure 4J**).

### 3.5 Combination of anlotinib and immunotherapy targets stromal cells to inhibit angiogenesis

**Figure 5:**
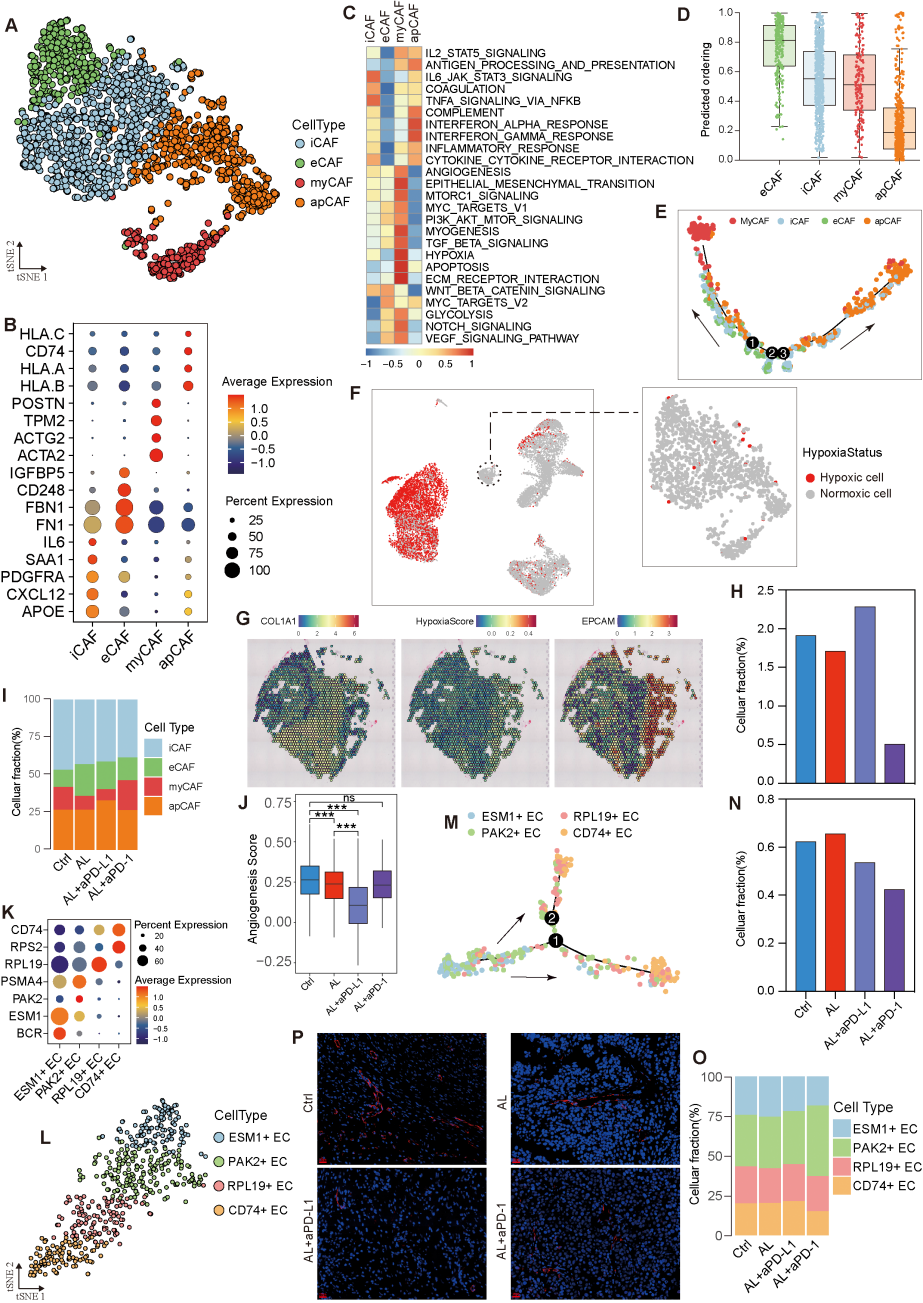
Heterogeneity and variation of stromal cells in the HGSOC TME. **(A)** t-SNE visualization of fibroblasts. **(B)** Dot plot depicting marker gene expression across fibroblast subtypes. **(C)** Enrichment analysis of fibroblast subtypes. **(D)** Analysis of differentiation levels in fibroblast subtypes. **(E)** Pseudotime trajectory analysis of epithelial subtypes. **(F)** Hypoxia status of fibroblasts. **(G)** Spatial transcriptome analysis confirming the hypoxia status of HGSOC cells and fibroblasts. **(H, I)** Variation in fibroblasts and fibroblast subtypes across different groups. **(J)** Angiogenesis capability across different groups. **(K)** Dot plot showing marker gene expression in endothelial subtypes. **(L)** t-SNE visualization of endothelial cells. **(M)** Pseudotime trajectory analysis of endothelial cell subtypes. **(N, O)** Variation in endothelial cells and endothelial cell subtypes across different groups. **(P)** Representative CD31 immunofluorescence (red) showing blood vessels in different groups. See also **Supplementary Figure S4**.

Cancer-associated fibroblasts (CAFs) are critical components of the TME, playing pivotal roles in tumor progression and treatment response. In this study, we identified six CAF clusters (Clusters 0–6) through dimensionality reduction analysis and marker genes expression (**Supplementary Figures S4A, B**). These clusters were categorized as follows: inflammatory CAFs (iCAFs), mainly associated with IL6-JAK-STAT3 signaling and TNFA-signaling-via-NFKB pathways (Clusters 0 and 2); myofibroblastic CAFs (myCAFs), expressing ACTA2, ACTG2, and POSTN, primarily involved in pathways such as angiogenesis, VEGF signaling, epithelial-mesenchymal transition (EMT), and hypoxia (Cluster 4); extracellular matrix CAFs (eCAFs), linked to WNT/β-catenin signaling and MYC targets (Cluster 3); and antigen-presenting CAFs (apCAFs), which involved in antigen presentation and the interferon response immune pathway (Clusters 5 and 1) (**Figures 5A–C**). Through pseudotime analysis, we identified seven distinct developmental states of CAFs (**Supplementary Figure S4C**). eCAFs exhibited the highest differentiation potential and were primarily located at the initial stages of development, possessing the ability to differentiate into myCAFs and apCAFs under specific conditions (**Figures 5D, E**, **Supplementary Figure S4D**). iCAFs were present across nearly all developmental stages, with genes such as CD24, CCL7, and PTEN influencing these developmental trajectories (**Supplementary Figure S4E, F**). Interestingly, we found that hypoxia within the TME mainly impacted tumor cells, while CAFs remained largely unaffected (**Figures 5F, G**). This observation raises the possibility that CAFs may maintain their oxygen supply by influencing angiogenesis, highlighting their potential role in TME angiogenesis. However, further experimental evidence is required to validate this hypothesis and elucidate the underlying mechanisms.

Following treatment with anlotinib or anlotinib + aPD-1, the proportion of CAFs decreased, whereas it increased in the anlotinib + aPD-L1 group (**Figure 5H**, **Supplementary Figure S4G**). Both anlotinib and anlotinib+ aPD-L1 significantly reduced the proportion of myCAFs, with the lowest levels observed in the anlotinib +aPD-L1 group (**Figure 5I**). Consequently, anlotinib + aPD-L1 treatment led to the lowest angiogenic capacity in CAFs, followed by anlotinib, with no significant changes observed in the anlotinib+ aPD-1 group (**Figure 5J**).

Endothelial cells (ECs) were divided into four sub-types based on gene expression profiles (**Figures 5K, L**), displaying varying levels of differentiation and distinct functional heterogeneity. ESM1^+^ ECs were capable of differentiating into PAK2^+^ ECs and RPL19^+^ ECs, eventually developing into CD74^+^ ECs (**Figure 5M**, **S4H**). ESM1^+^ ECs were highly enriched in angiogenesis-related pathways—such as VEGF, PI3K-AKT-mTOR, TGFβ, and coagulation signaling—endowing them with the strongest angiogenic capacity. In contrast, CD74^+^ ECs showed the lowest enrichment in these pathways, resulting in the weakest angiogenic capacity (**Supplementary Figures S4I, J**). In both the anlotinib + aPD-L1 and anlotinib + aPD-1 groups, the proportion of endothelial cells decreased, particularly the ESM1^+^ ECs, leading to the decreasing angiogenic capacity of endothelial cells (**Figures 5N, O**, **Supplementary Figure S4K**). Immunofluorescence staining showed that anlotinib and anlotinib + aPD-L1 treatment significantly reduced blood vessel numbers and made vessel structures more regular (**Figure 5P**).

In conclusion, anlotinib appears to inhibit angiogenesis in the TME primarily by reducing the proportion of myCAFs. The addition of aPD-L1 enhances this effect, as evidenced by a further reduction in the proportions of myCAFs and ESM1^+^ ECs, thereby significantly limiting angiogenesis and affecting the oxygen supply to tumor cells. While CAFs seem unaffected by hypoxia in this context, the mechanisms underlying their oxygen supply and potential preferential access to vascular resources warrant further investigation.

### 3.6 Treatment effects on T cell subtypes: reversal of exhaustion and enhanced recruitment

To further assess the impact of anlotinib combined with aPD-L1/aPD-1 on tumor-infiltrating lymphocytes (TILs) within the TME, we conducted an in-depth analysis of T cells.

**Figure 6:**
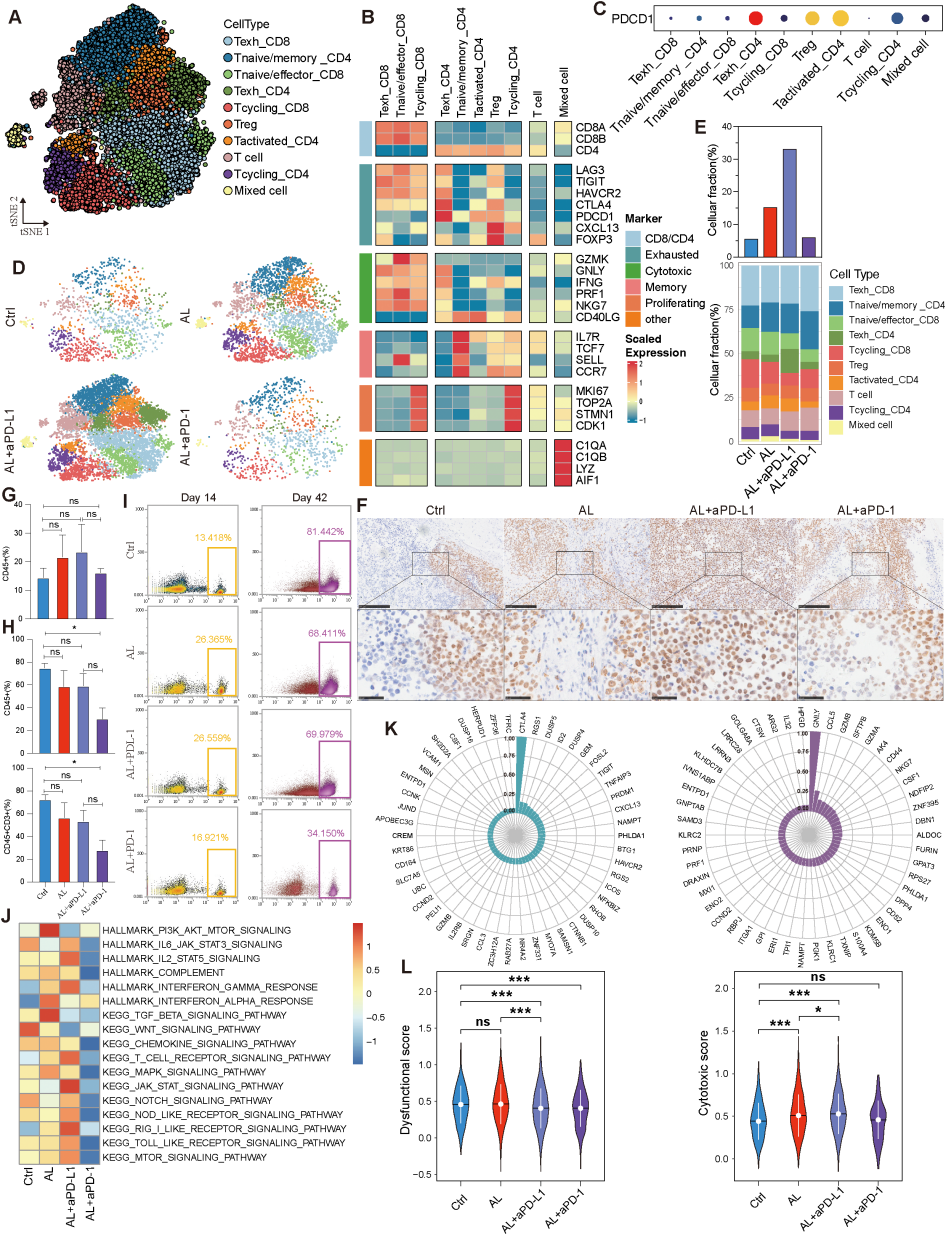
T lymphocytes variation across different treatment groups. **(A)** t-SNE visualization of T lymphocytes. **(B)** Expression levels of status-associated genes across T lymphocyte subtypes. **(C)** Expression of PDCD1 in T lymphocyte subtypes. **(D, E)** Variation in T lymphocytes and their subtypes across different groups. **(F)** Immunohistochemistry of CD8A across different groups. **(G)** Variation in CD45+ cells across different groups at day 14. **(H)** Variation in CD45+ and CD45+CD3+ cells across different groups at day 42. **(I)** Variation in CD45+ cells across different groups at days 14 and 42. **(J)** Immune-related pathway enrichment analysis across different groups. **(K)** Pearson correlation analysis of the top 50 genes related to the dysfunctional gene CTLA4 and the cytotoxic gene GNLY. **(L)** Comparison of dysfunctional and cytotoxic scores across different groups. See also **Supplementary Figure S5**.

Based on specific marker expression, T cells were categorized into ten distinct subtypes, predominantly expressing either CD8A or CD4 genes, while a subset lacking CD4, CD8, and other specific markers but expressing CD3D and CD3E was defined as “T cell” (**Figure 6A**, **Supplementary Figure S5A**). Among the CD4^+^ cells, we identified subtypes including exhausted helper T cells (Texh_CD4), regulatory T cells (Treg), naive/memory T cells (T naive/memory_CD4), activated T cells (Tactivated_CD4), and cycling T cells (Tcycling_CD4) (**Figure 6B**). T cycling_CD4 cells exhibited strong proliferative and differentiation potential, capable of maturing into other CD4^+^ subtypes. In contrast, Texh_CD4 cells were at a terminal differentiation stage, exhibiting progressively exhausted functions (**Supplementary Figures S5B, C**). Within the CD8^+^ T cell population, we identified three subtypes: exhausted cytotoxic T cells (Texh_CD8), naive/effector cytotoxic T cells (Tnaive/effector_CD8), and proliferating T cells (Tcycling_CD8) (**Figure 6B**). Among these, Tcycling_CD8 cells can differentiate into Tnaive/effector_CD8 cells. Under conditions of chronic antigen exposure, these effector cells may eventually transition into terminally exhausted Texh_CD8 cells (**Supplementary Figures S5D, E**). This developmental trajectory aligns with the differentiation patterns and biological roles of various T cell subtypes, with exhausted cells representing a dysfunctional state characterized by reduced effector functions and altered responsiveness (30, 31).

Strangely, PD-1 (PDCD1) expression was relatively low in Texh_CD8 cells, despite high expression of other exhaustion markers such as LAG3, TIGIT, and HAVCR2. In contrast, Texh_CD4, Treg, and Tactivated_CD4 cells exhibited higher levels of PD-1 expression (**Figures 6B, C**), while HGSOC tumor cells displayed low PD-L1 (or CD274) expression level (**Supplementary Figures S5F, G**). Additionally, we observed an increase in T lymphocytes within the TME following treatment, with the most pronounced elevation in the anlotinib + aPD-L1 group, whereas the anlotinib + aPD-1 group demonstrated a more modest rise (**Figures 6D, E**). Immunohistochemistry confirmed that the anlotinib + aPD-L1 group exhibited higher levels of CD8^+^ TILs, a factor closely associated with the efficacy of immunotherapy (**Figure 6F**). To investigate the cause of the differential TIL levels, we analyzed peripheral blood from the PBMC-PDX models. At the onset of treatment, all three treatment groups exhibited higher levels of CD45^+^ immune cells compared to control group (**Figures 6G, I**). However, by the end of treatment, the proportions of both CD45^+^ and CD3^+^ cells were lower across all treatment groups than in controls (**Figure 6H, I**, **Supplementary Figure S5H**). The observed decrease in peripheral blood immune cells, coupled with the increase in TME T cells, strongly suggests T cell migration from peripheral blood to the TME, consistent with the characteristics of the PBMC-PDX model.

The efficacy of ICIs is closely related not only to the quantity of CD8^+^ TILs but also to their functional state. We found that treatment with anlotinib or anlotinib + aPD-L1 decreased the proportion of Texh_CD8 cells, whereas the Texh_CD8 population increased in the anlotinib + aPD-1 group (**Figure 6E**). Furthermore, both anlotinib and anlotinib + aPD-L1 treatments significantly enhanced the inflammatory response within the TME and promoted immune activation (**Figure 6J**). To further validate this observation, we analyzed the top 50 genes associated with the exhaustion marker CTLA4 and the cytotoxic marker GNLY, calculating and comparing the exhaustion and cytotoxic scores of CD8^+^ TILs (**Figure 6K**). The analysis revealed that both dysfunctinal and cytotoxic score increased in the anlotinib treatment group, indicating that anlotinib promotes the infiltration of CD8^+^ T cells into the TME—including both exhausted and non-exhausted cells—but does not reverse the exhausted state of existing Texh_CD8^+^ cells (**Figure 6L**). In contrast, dysfunctional score decreased and cytotoxic score increased in the anlotinib + aPD-L1 (**Figure 6L**), suggesting the reactivation of exhausted CD8^+^ TILs. Additionally, following anlotinib + aPD-L1 treatment, the Texh_CD8 subtype exhibited an increase in downregulated genes, while the Tnaive/effector_CD8 subtype showed a significant increase in upregulated genes, further supporting the potential reversal of CD8^+^ T cell exhaustion (**Supplementary Figure S5I**). Moreover, IF results demonstrated that the number and proportion of CD8^+^GZMB^+^ T cells were higher in the anlotinib + aPD-L1 group compared to both the control and anlotinib monotherapy groups (**Supplementary Figure S6A–C**).

Summarily, anlotinib increases TIL levels, establishing a foundation for improved ICI efficacy. The combination of anlotinib + aPD-L1 achieved superior therapeutic outcomes by facilitating stronger T cell recruitment and exhausted CD8^+^ T cell reactivation.

### 3.7 Combination of anlotinib and immunotherapy induced modulation of myeloid cell polarization and anti-tumor activity

**Figure 7:**
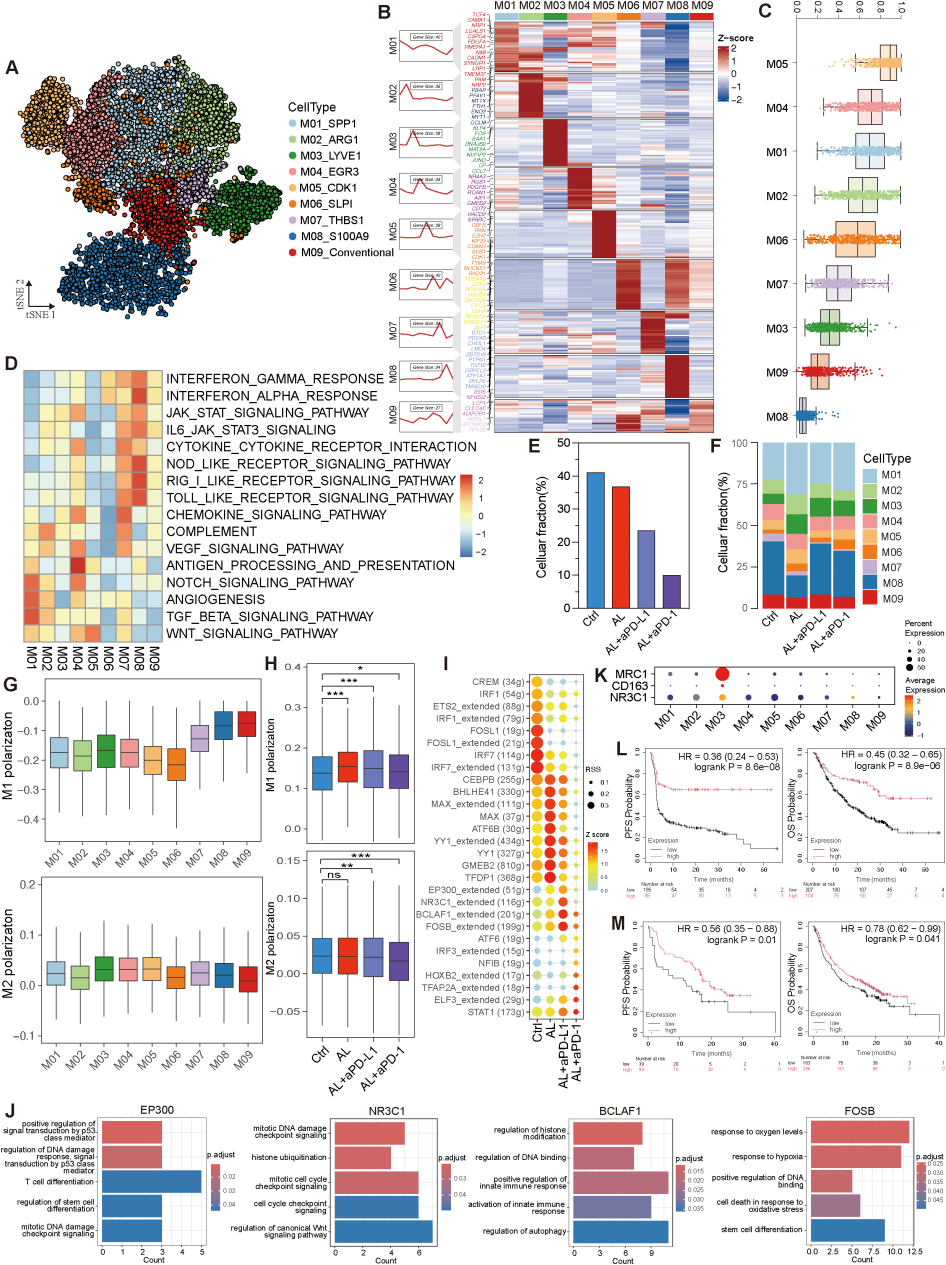
Variation of myeloid cells after treatments in HGSOC TME. **(A)** t-SNE visualization of myeloid cell subtypes. **(B)** Expression profile of myeloid cell subtypes. **(C)** Analysis of differentiation levels in myeloid cell sub-types. **(D)** Pathway enrichment analysis of myeloid cell subtypes. **(E, F)** Variation in myeloid cells and myeloid cell subtypes across different groups. **(G)** M1 and M2 polarization of myeloid cell subtypes. **(H)** Comparison of M1 and M2 polarization of myeloid cells across different groups. **(I)** Activity and regulon specificity scores of transcription factors across different groups. **(J)** GO BP enrichment of transcription factor in the anlotinib + aPD-L1 group. **(K)** Co-expression of NR3C1 with CD163 and MRC1. **(L, M)** Kaplan-Meier plots showing the correlation between NR3C1 expression and progression-free survival (PFS) and overall survival (OS) in cancer patients treated with aPD-1 **(L)** and aPD-L1 **(M)**, with data sourced from the Kaplan-Meier Plotter database. See also **Supplementary Figure S7**.

Myeloid cells constitute another significant component within the TME. We categorized these myeloid cells into nine distinct subtypes based on dimensionality reduction clustering and gene expression profiling (**Figures 7A, B**). Except for M09, all other subtypes expressed macrophage markers such as CD68, C1QB, S100A9, and AIF1, suggesting that they are tumor-associated macrophages (TAMs) (**Supplementary Figure S7A**). Among these, M01_SPP1 and M08_S100A9 were the most prevalent (**Supplementary Figure S7B**). These subtypes exhibited varying levels of differentiation. M05_CDK1, which demonstrated the highest proliferative and differentiation potential, occupied the starting point of the developmental trajectory and had the ability to differentiate into various subtypes (**Figure 7C**, **Supplementary Figure S7C**). This differentiation followed multiple developmental branches, highlighting the significant heterogeneity within the myeloid population. M08_S100A9, with the lowest differentiation potential, was positioned at the terminal stage of these trajectories (**Figure 7C**, **Supplementary Figure S7D**). Moreover, these subtypes exhibited distinct biological roles: M01 was associated with angiogenesis, M04 was primarily involved in antigen presentation, and M07, M08, and M09 were enriched in immune and inflammation-related pathways (**Figure 7D**).

Following treatment, the proportion of myeloid cells decreased, particularly in the anlotinib + aPD-L1 group, coupled with an increase in M01 cells. Both the anlotinib and anlotinib + aPD-1 groups saw a significant rise in the proportion of M01 cells, enhancing the angiogenic capacity of tumor-associated macrophages (TAMs). In contrast, the M01 population remained relatively stable in the anlotinib + aPD-L1 group, along with a diminished angiogenic potential (**Figures 7E, F**, **Supplementary Figure S7E**).

To further delineate the inflammatory states of myeloid cells after different treatments, we calculated M1 and M2 polarization scores using specific gene sets. The data indicated that M07, M08, and M09 exhibited higher M1 polarization scores, while M03 had the highest M2 polarization score (**Figure 7G**). After treatment, M1 polarization increased, with the most notable rise in the anlotinib group, whereas M2 polarization scores decreased in the anlotinib + aPD-L1 and anlotinib + aPD-1 groups (**Figure 7H**). These treatments also enhanced antigen-presenting capability and inhibited immune escape (**Supplementary Figure S7E**), indicating a promotion of pro-inflammatory and anti-tumor activity within the myeloid cells in the TME. Additionally, myeloid cells displayed more upregulated than downregulated genes after anlotinib + aPD-L1 treatment, with the M01 subtype showing the most prominent changes, suggesting that the treatment may regulate myeloid cells to promote pro-inflammatory and anti-tumor effects (**Supplementary Figure S7F**).

Additionally, these therapies induced significant changes in transcription factor regulon activity. In the anlotinib + aPD-L1 group, the activity of regulons such as EP300, NR3C1, BCLAF1, and FOSB, which participate in DNA damage response, immune regulation, stress adaptation mechanisms, and response to hypoxia, were notably elevated (**Figures 7I, J**). Interestingly, NR3C1 was co-expressed with MRC1 and CD163 in M03 cells (**Figure 7K**), suggesting that NR3C1 may play a role in inhibiting immune-mediated tumor cell killing. We further analyzed NR3C1 in pan-cancer patients receiving aPD-L1/aPD-1 therapy and found that NR3C1 expression was positively correlated with both PFS and OS (**Figures 7L, M**). This discrepancy may stem from a shift in NR3C1’s role after immunotherapy.

In conclusion, anlotinib treatment enhances the tumor-killing ability of immune cells and suppresses immune escape via regulating myeloid cells, while the combination of anlotinib with aPD-L1 or aPD-1 further amplifies these anti-tumor effects.

### 3.8 Alterations in cell-to-cell communication networks after anlotinib-based treatments

**Figure 8:**
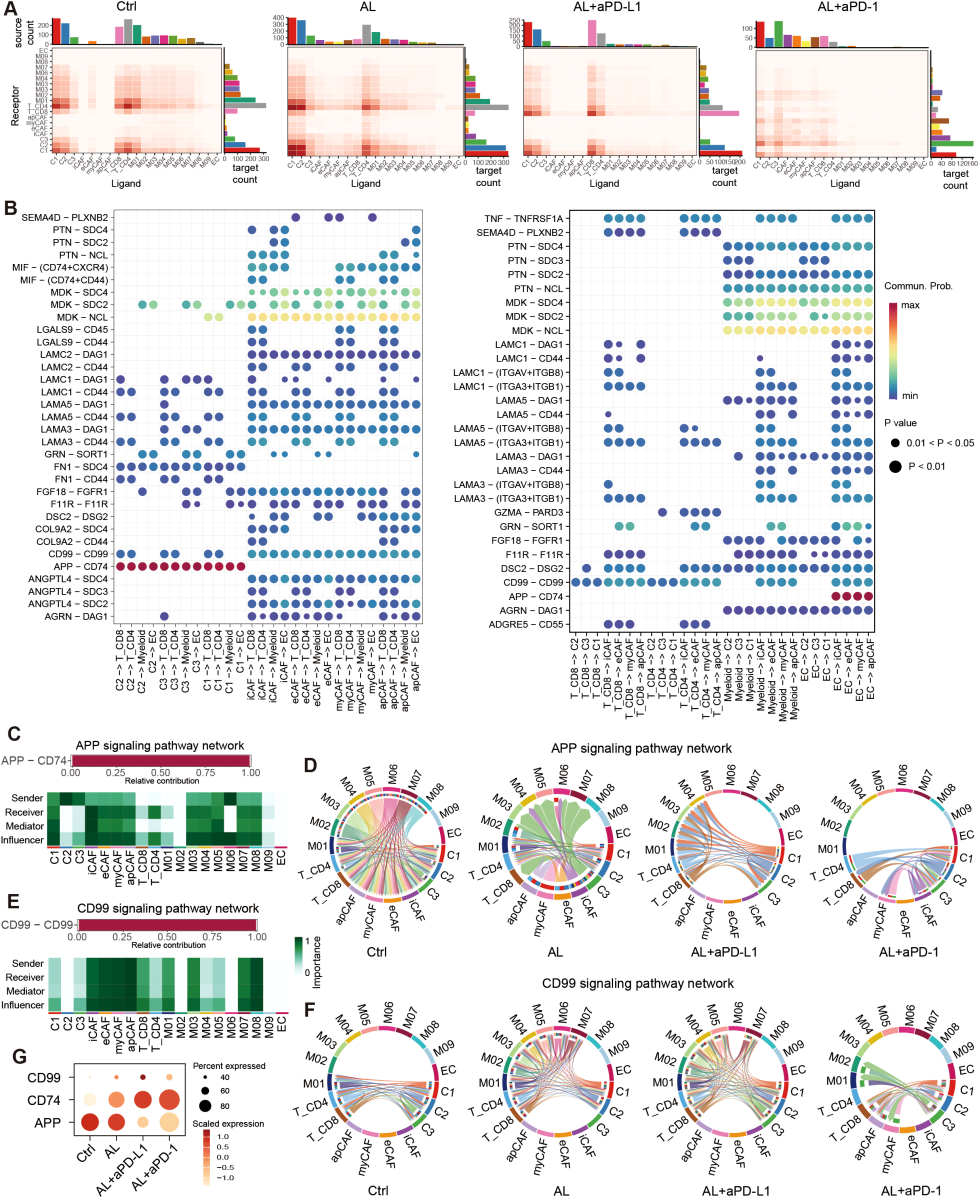
Cell-cell communication in the HGSOC TME. **(A)** Heatmap showing the number of total potential ligand–receptor pairs between different cell types in each group obtained with CellChat. The bar plot represents the sum of a column or row. **(B)** Dot plot showing the main significant ligand-receptor pairs between tumor cells, fibroblast and T cells, myeloid cells, endothelial cells. The dot color and size represent the calculated communication probability and p-values. p-values are computed from a one-sided permutation test. **(C)** The L-R paired APP signaling include, and the relative importance of cell types in APP signaling. **(D)** Chord plot showing the variation of APP signaling in different groups. **(E)** The L-R paired CD99 signaling include, and the relative importance of cell types in CD99 signaling. **(F)** Chord plot showing the variation of CD99 signaling in different groups. **(F)** Dot plot showing the expression of APP, CD74, CD99. See also **Supplementary Figure S8**.

To investigate alterations in intercellular communication signals following treatment, we conducted a comprehensive cell-to-cell communication analysis. Compared to the control group, treatment with anlotinib led to an increased number of cell communications; however, the intensity of these interactions was reduced. This reduction may be attributed to decreased activity in certain ligand–receptor pairs (**Supplementary Figure S8A**). Notably, anlotinib promoted increased communication between CAFs and tumor cells, while interactions between CD8^+^ T cells and other cell types diminished (**Figure 8A**). In contrast, after treatment with anlotinib + aPD-L1, although the overall number and intensity of cell communications decreased further, communication involving CD8^+^ T cells increased significantly, which didn’t happen when treated with anlotinib + aPD-1 (**Figure 8A**).

We further identified that the APP–CD74 ligand–receptor pair (LRP) primarily mediated communication between tumor cells and immune cells. Additionally, MDK–NCL/SDC2/SDC4 interactions played a central role in CAF interactions with other cells, while CD99–CD99 interactions were widely involved in communications between immune cells and tumor cells (**Figure 8B**, **Supplementary Figure S8B**). These LRPs form the core components of the APP, CD99, and MK signaling pathways, respectively (**Figures 8C, E**, **Supplementary Figure S8C**).

Following treatment, the number of MK signaling interactions remained relatively unchanged. However, communication through the APP signaling pathway was significantly reduced (**Supplementary Figure S8D**). In the APP pathway, tumor cells primarily acted as signal senders, with immune cells (T cells and myeloid cells) serving as primary receivers, and CAFs also playing a vital role (**Figure 8C**). After treatment with anlotinib, signal output from myeloid cells decreased, and CAFs transitioned from being signal receivers to signal senders. Following treatment with anlotinib combined with aPD-L1 or aPD-1, APP signaling was further diminished, while interactions between CD8^+^ T cells and tumor cells increased (**Figure 8D**). Conversely, the CD99 signaling pathway was significantly upregulated after treatment (**Supplementary Figure S8D**). Although CAFs contributed to this pathway, their involvement in communications was relatively minor (**Figure 8E**). In the control group, CD99 signaling predominantly occurred between CD4^+^ and CD8^+^ T cells and tumor cells. After anlotinib treatment, interactions among M01–M07 myeloid cells, CD4^+^ and CD8^+^ T cells, and C1 and C2 tumor cells increased notably.

This trend persisted in the anlotinib + aPD-L1 group. However, the changes in CD99 signaling were less pronounced in the anlotinib+ aPD-1 group (**Figure 8F**). These results suggest that anlotinib and its combination with aPD-L1 may enhance CD99 signaling between myeloid cells, T cells, and tumor cells, thereby improving immune-mediated tumor destruction and leading to better therapeutic outcomes. The observed changes in APP, CD74, and CD99 expression across different treatment groups further support these findings (**Figure 8G**).

## 4 Discussion

In this study, we systematically evaluated the potential efficacy of the anlotinib, and its combination with aPD-1/aPD-L1 in the treatment of HGSOC. Through clinical patient data, HGSOC derived PBMC-PDX models, and scRNA analysis, we found that anlotinib monotherapy exhibited anti-tumor effects, while the combination with aPD-L1 significantly enhanced anti-angiogenic effects, improved T-cell infiltration, and effectively reversed immune evasion. These findings are visually summarized in **Supplementary Figure S9**, which illustrates the dynamic changes in key cell populations within the TME. This combination strategy offers a new approach for HGSOC treatment, especially in situations where the current immune therapies show limited efficacy, and thus displays broad clinical potential.

From real-world data that anlotinib monotherapy achieved a 71.43% DCR and a median PFS of 6.5 months in patients with recurrent or refractory HGSOC. In particular, the combination with aPD-L1/aPD-1 achieved a DCR of 100%, with a prolonged PFS of 8.7 months. The combination of anlotinib and aPD-L1/aPD-1 has demonstrated synergistic antitumor effects across various cancer types (28, 32), even in other refractory cancers and triple-negative breast cancer, which is also classified as “cold tumor” (33, 34). While limited studies have reported on the efficacy and safety of this combination therapy in HGSOC, conclusive results and in-depth analyses remain lacking (35), and several clinical trials are still ongoing (36). These has contributed to the fact that anlotinib combination with aPD-L1/aPD-1 have not yet been approved for use in HGSOC.

We further validated the effects of the treatment by constructing PBMC-PDX models. The results showed that the combination of anlotinib and aPD-L1 significantly delayed tumor growth, exhibiting more pronounced anti-tumor effects compared to monotherapy. ScRNA also confirmed that anlotinib + aPD-L1 further reduced the proportion of tumor cells compared to anlotinib alone.

Regarding the mechanisms, anlotinib treatment increased hypoxia and apoptosis in tumor tissue while reducing the proportion of proliferating cells. Additionally, it decreased the expression and activity of the proliferation-associated E2F transcription factor, which is primarily linked to cell cycle regulation and tumor proliferation. These findings suggest that anlotinib inhibits both tumor cell proliferation and angiogenesis, aligning with observations from previous studies (9, 37). Interestingly, we found that aPD-L1 enhanced anlotinib’s pro-apoptotic effects and further increased hypoxia levels, as indicated by elevated expression and activity of ATF4 and ATF6, transcription factors associated with hypoxic conditions (38). This intensified hypoxia may result from increased immune cell infiltration and activation, leading to greater oxygen consumption or immune-mediated vascular disruption, thereby reducing oxygen supply (39, 40).

In the present study, we found that anlotinib’s inhibition of angiogenesis was primarily associated with myCAFs, a subtype originally identified in pancreatic ductal adenocarcinoma but less studied in HGSOC (41–44). The reduction in myCAF populations following anlotinib or anlotinib + aPD-L1 treatment significantly impaired HGSOC’s angiogenic capacity, leading to pronounced hypoxia in tumor cells. Interestingly, while tumor cells experienced hypoxia after treatment, CAFs did not, suggesting that CAFs may maintain their oxygen supply by modulating blood vessel formation, possibly through localized angiogenesis or spatial proximity to functional vasculature.

Endothelial cells (ECs) are another major target of anlotinib (45). Specifically, we identified ESM1^+^ ECs as the primary endothelial cell population driving angiogenesis in the HGSOC TME, with their functional decline closely associated with reduced angiogenic capacity after treatment. Conversely, the proportion of CD74^+^ ECs increased following combination therapy, potentially indicating a shift in endothelial cell states that further inhibits angiogenesis.

In addition to its effects on tumor and stromal cells, anlotinib also improved the immunosuppressive microenvironment by significantly increasing T cell and CD8^+^ T cell infiltration, likely achieved through vascular normalization. Anlotinib’s anti-angiogenic properties reduce abnormal tumor vasculature and promote vascular normalization, thereby alleviating high interstitial fluid pressure and increasing vascular permeability. These changes facilitate the migration of immune cells, particularly cytotoxic CD8^+^ T cells, into the TME, as similarly reported in studies on lung cancer and hepatocellular carcinoma (46–48).

However, anlotinib monotherapy was unable to reverse the exhausted state of CD8^+^ T cells. In contrast, anlotinib combined with aPD-L1 not only enhanced CD8^+^ T cell infiltration but also improved the functional state of these exhausted cells, suggesting that this combination therapy enhances both the quantity and cytotoxic activity of CD8^+^ T cells, making them more effective at eliminating tumor cells. This indicates that while vascular normalization facilitates immune cell infiltration into the tumor microenvironment, the reactivation of exhausted T cells primarily depends on immune checkpoint blockade, such as aPD-L1, rather than on vascular normalization alone. Furthermore, VEGF/VEGFR-targeted therapy not only reduces abnormal vasculature but also alleviates the immunosuppressive effects of VEGF, creating a microenvironment conducive to immune cell activity, while PD-L1 blockade directly restores T cell effector functions (49, 50). These complementary mechanisms underlie the synergistic effect of the combination therapy.

Conversely, anlotinib combined with aPD-1 did not produce a similar effect on T cells. Our analysis showed that PD-1 expression was primarily on Tregs and low on CD8^+^ T cells, suggesting that aPD-1 therapy may preferentially target PD-1-expressing Tregs. This interaction could enhance the suppressive activity of Tregs, potentially contributing to the limited efficacy observed with anlotinib and aPD-1, thus explaining why the anlotinib and aPD-L1 combination demonstrated superior outcomes (51–53).

Recent studies align with our findings, supporting the efficacy of combined anti-angiogenic and immune checkpoint therapies. For instance, the Phase 3 IMpower150 trial demonstrated that atezolizumab combined with bevacizumab/chemotherapy achieved strong anticancer activity and manageable side effects in NSCLC (54). Similarly, the combination of anti-angiogenic therapy and immunotherapy showed superior tumor reduction in renal cell carcinoma and hepatocellular carcinoma (47, 55). Sequential therapy was also explored in NSCLC, where initiating anti-angiogenic therapy after immune checkpoint blockade showed better outcomes than concurrent or reverse sequencing (56). Mechanistically, anti-angiogenic therapy normalizes tumor vasculature, improving cytotoxic TILs and reducing interstitial fluid pressure. This normalization enhances immune cell access to the TME, creating conditions favorable for ICIs to reactivate exhausted T cells and boost antitumor immunity (57–59). Additionally, anti-angiogenic therapies downregulate immunosuppressive factors and reverse endothelial cell deactivation, further augmenting the effects of ICIs (60).

Myeloid cells also exhibited substantial changes post-treatment. Anlotinib, alone or in combination with aPD-L1/aPD-1, significantly increased the M1 polarization score while reducing the M2 polarization score when combined with aPD-L1/aPD-1. Additionally, anlotinib or its combinations enhanced myeloid cells’ antigen-presenting capability and inhibited immune escape. These immunomodulatory effects on both T cells and myeloid cells indicate that anlotinib can reshape the immunosuppressive TME in HGSOC, potentially improving responsiveness to aPD-L1. Study in neuroblastoma demonstrated that anlotinib could reprogram an immunosuppressive TME into an immunostimulatory environment, curbing tumor growth and preventing systemic immunosuppression, while in lung cancer, it was shown to inhibit M2 polarization of TAMs through the AKT/mTORC1 and Pparδ pathways (61, 62).

Further analysis suggested that the effects of anlotinib, both alone and combined with aPD-L1, on the immune microenvironment may be mediated through inhibition of APP signaling and enhancement of CD99 signaling. APP signaling transmits inhibitory signals that suppress the phagocytic activity of tumor-associated macrophages (TAMs) (63) and reduces immune activity within the TME, allowing tumor cells to evade immune surveillance and promote tumor progression (64, 65). In contrast, CD99 signaling may enhance T cell cytotoxicity and induce tumor cell apoptosis (66, 67). Given the limited studies on APP-CD74 and CD99-CD99 signaling in HGSOC, further research is required to clarify their specific roles in the TME.

In conclusion, this study demonstrated the therapeutic potential of anlotinib combined with aPD-L1/aPD-1 in HGSOC. Anlotinib combination with aPD-L1 effectively inhibits angiogenesis, suppresses tumor cell proliferation, and transforms cold tumors into hot tumors by enhancing TIL infiltration, reactivates exhausted TILs, and fosters a tumor-killing environment, showing strong synergistic effects compare to anlotinib monotherapy. To our knowledge, this is the first study to utilize scRNA-seq to evaluate the efficacy and underlying mechanisms of anlotinib combined with aPD-L1/aPD-1 therapy in HGSOC. These findings lay a promising foundation for advancing precision treatment strategies in refractory HGSOC.

Although neither anlotinib nor aPD-L1 is currently approved for HGSOC, the manageable toxicity and preclinical efficacy observed suggest that this therapy could be safely implemented in clinical trials, which has been implemented in other solid tumors recently (48, 68–70). Based on our findings and relevant reports, potential biomarkers for patient stratification include PD-L1 expression, TMB levels, the CD8+ TIL/Treg ratio, the M1/M2 myeloid cell ratio, and tertiary lymphoid structures, all of which reflect an immunostimulatory TME (68). Future clinical trials could incorporate these biomarkers to identify subgroups most likely to benefit, to advance precision treatments for refractory HGSOC.

While this study provides promising insights, several limitations remain.

First, the small sample size (n=36) limits the generalizability of the findings, large-scale clinical trials are essential for further validation. We will continue to collect relevant cases to increase the reliability of the results. Second, this study utilized a single PDX model to evaluate the effects of anlotinib combined with immunotherapy, which limits the generalizability of the findings. Additionally, the small sample size may introduce statistical bias, and the lack of functional immune assays, such as cytotoxicity tests or cytokine secretion analysis, limits the mechanistic understanding of immune responses. Furthermore, the absence of longitudinal measurements precludes capturing the dynamic changes during treatment. To address these limitations, we are constructing additional PDX models from diverse HGSOC patient samples, incorporating functional assays like cytotoxicity tests and cytokine profiling, and designing longitudinal studies to monitor treatment effects over time. These efforts aim to enhance the robustness and translational potential of our findings.

Additionally, although we demonstrated the therapeutic efficacy of anlotinib combined with aPD-L1, the combination of anlotinib with aPD-1 showed comparatively poorer outcomes. This discrepancy between the effects of AL + aPD-1 and AL + aPD-L1 prompted us to further investigate the underlying mechanisms. Our findings revealed several potential explanations: (1) PD-1 (PDCD1) expression was predominantly localized to Tregs and CD4^+^ T cells, and the use of aPD-1 may have inadvertently enhanced Treg-mediated immunosuppression, contributing to immune evasion. (2) AL + aPD-1 was less effective than AL + aPD-L1 in promoting M1 polarization of myeloid cells, though the precise mechanism remains unclear. (3) AL + aPD-1 demonstrated a reduced ability to recruit T cells into the TME compared to AL + aPD-L1, potentially due to broader immunoregulatory effects of aPD-L1 on TME composition. These findings suggest that PD-1 and PD-L1 inhibitors might exert distinct regulatory effects on immune cells within the TME, resulting in differential therapeutic outcomes.

Several important questions remain to be addressed: Is this unique pattern of PD-1 (PDCD1) expression (predominantly in Tregs and CD4^+^ T cells) a common feature in HGSOC? Could this expression pattern underlie the poor response of HGSOC to immune checkpoint inhibitors? Addressing these questions in future studies will help to clarify the role of PD-1 and PD-L1 pathways in modulating the immune microenvironment of HGSOC and guide the development of more effective therapeutic strategies.

Moreover, addressing resistance remains another critical challenge for future investigations. Tumors may adapt to anti-angiogenic therapy by upregulating alternative pro-angiogenic pathways or through ECM deposition, which hinders immune cell infiltration and drug delivery (71, 72). For ICIs, resistance mechanisms include T cell exhaustion, alternative immune checkpoints, or immunosuppressive activity from Tregs, MDSCs, and TAMs (73). Overcoming these challenges may require combining therapies targeting compensatory angiogenic pathways, hypoxia-related factors, or sequential immune checkpoint blockades. Additionally, given that platinum-based chemotherapy remains the standard treatment for HGSOC and most anlotinib use in this study occurred after ≥3 lines of prior treatment, future research will focus on evaluating concurrent or sequential combinations of chemotherapy, anti-angiogenic therapy, and immunotherapy to optimize strategies and improve patient outcomes.

## Data Availability

The scRNA-seq data presented in the study are deposited in the Genome Sequence Archive repository (https://ngdc.cncb.ac.cn/gsa), accession number CRA024166.
