# Supplementary material for: Combination of anlotinib with immunotherapy enhanced both anti-angiogenesis and immune response in high-grade serous ovarian cancer
Source: Front Immunol. 2025 Apr 7;16:1539616. doi: 10.3389/fimmu.2025.1539616 (PMC12009696; doi:10.3389/fimmu.2025.1539616)
Supplement: Supplementary file 2 [file DataSheet2.zip › Supplementary_Material_Revised.docx]

**Figure S1. Flow chart of the retrospective study of anlotinib-based therapy in patients with treatment-refractory high-grade serous ovarian cancer (HGSOC)**


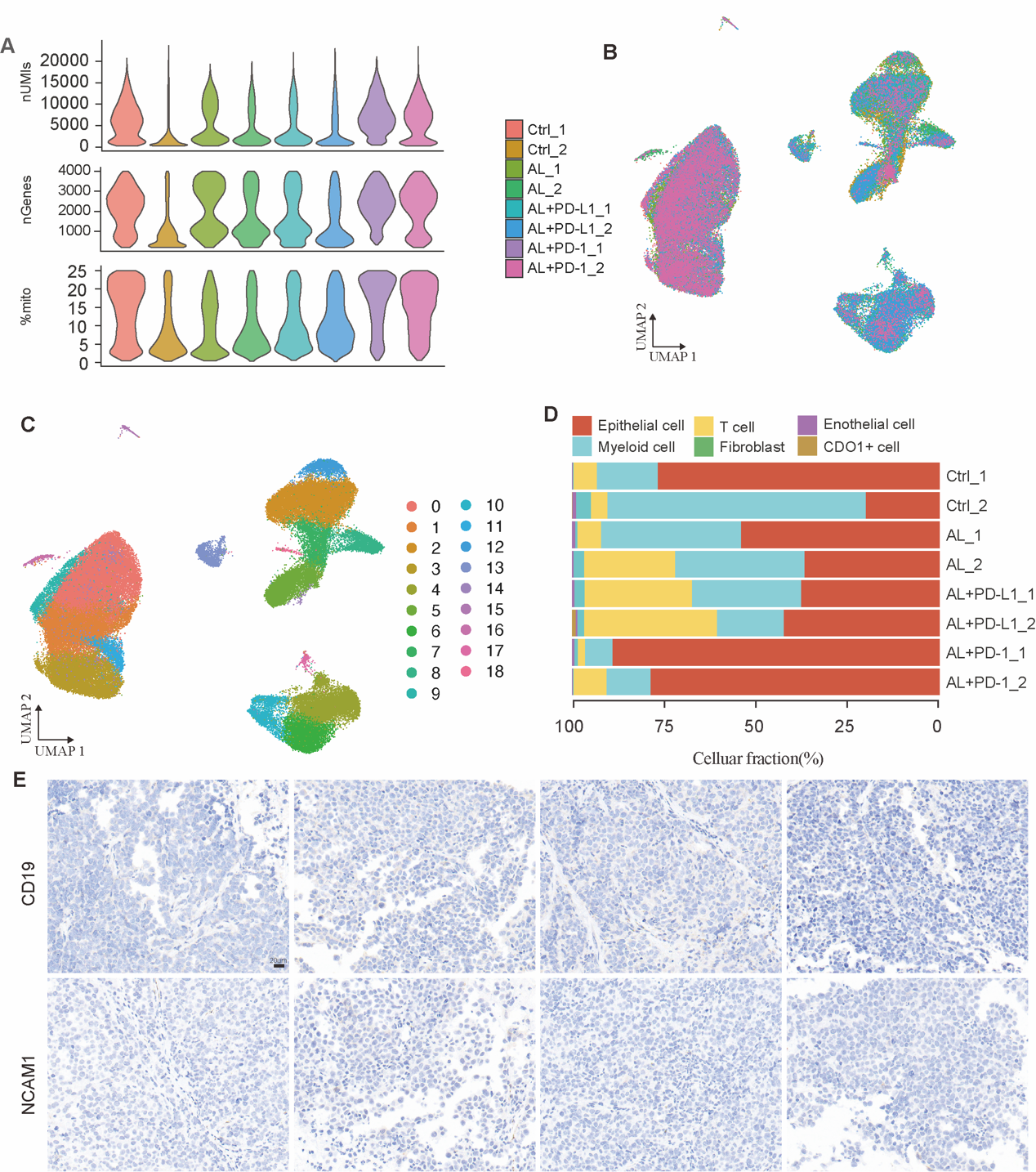


**Figure S2. Quality Control and Cell Type Identification in the HGSOC TME**

(A) Violin plots displaying the distributions of the number of unique molecular identifiers (UMIs) detected (top), the number of genes detected (middle), and the percentage of mitochondrial gene content (bottom) per cell after quality control across groups. (B) UMAP visualization of cell distribution across samples. (C) UMAP visualization displaying all cell clusters. (D) Proportions of the five major cell types across all samples. (E) IHC images showing the expression of CD19 and NCAM1, which are markers for B cells and NK cells, respectively.


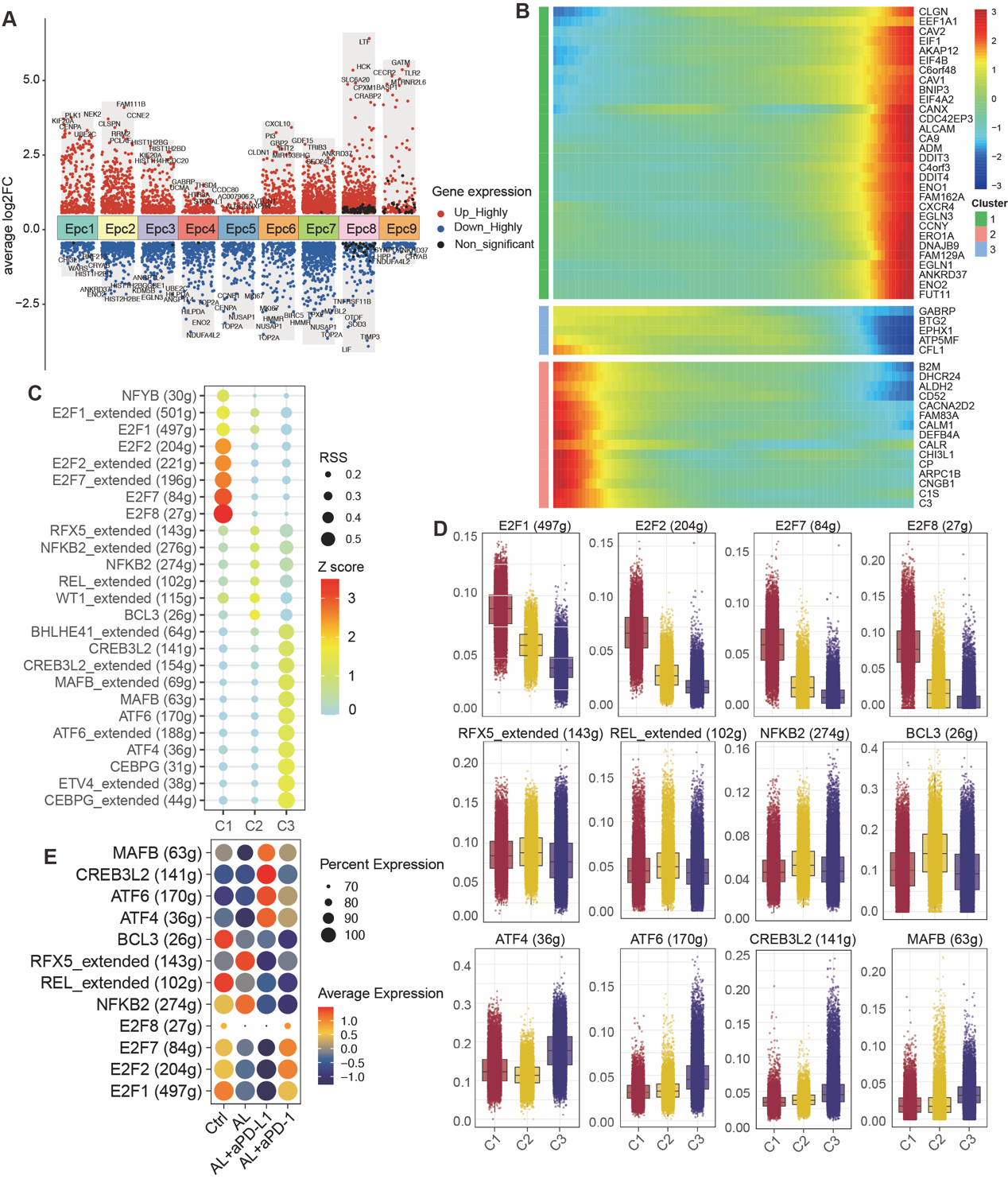


**Figure S3. Heterogeneity and Variation of Epithelial Cells in the HGSOC TME**

(A) Differentially expressed genes (DEGs) in Epc1-Epc9 epithelial cell subtypes. (B) Hierarchical clustering heatmap displaying three subclusters of the top 50 genes along the pseudo-time trajectory of epithelial cells. (C) Activity and regulon specificity scores of transcription factors in C1, C2, and C3 epithelial subtypes. (D) Expression of key transcription factors in C1, C2, and C3 epithelial subtypes. (E) Expression of key transcription factors across different groups.


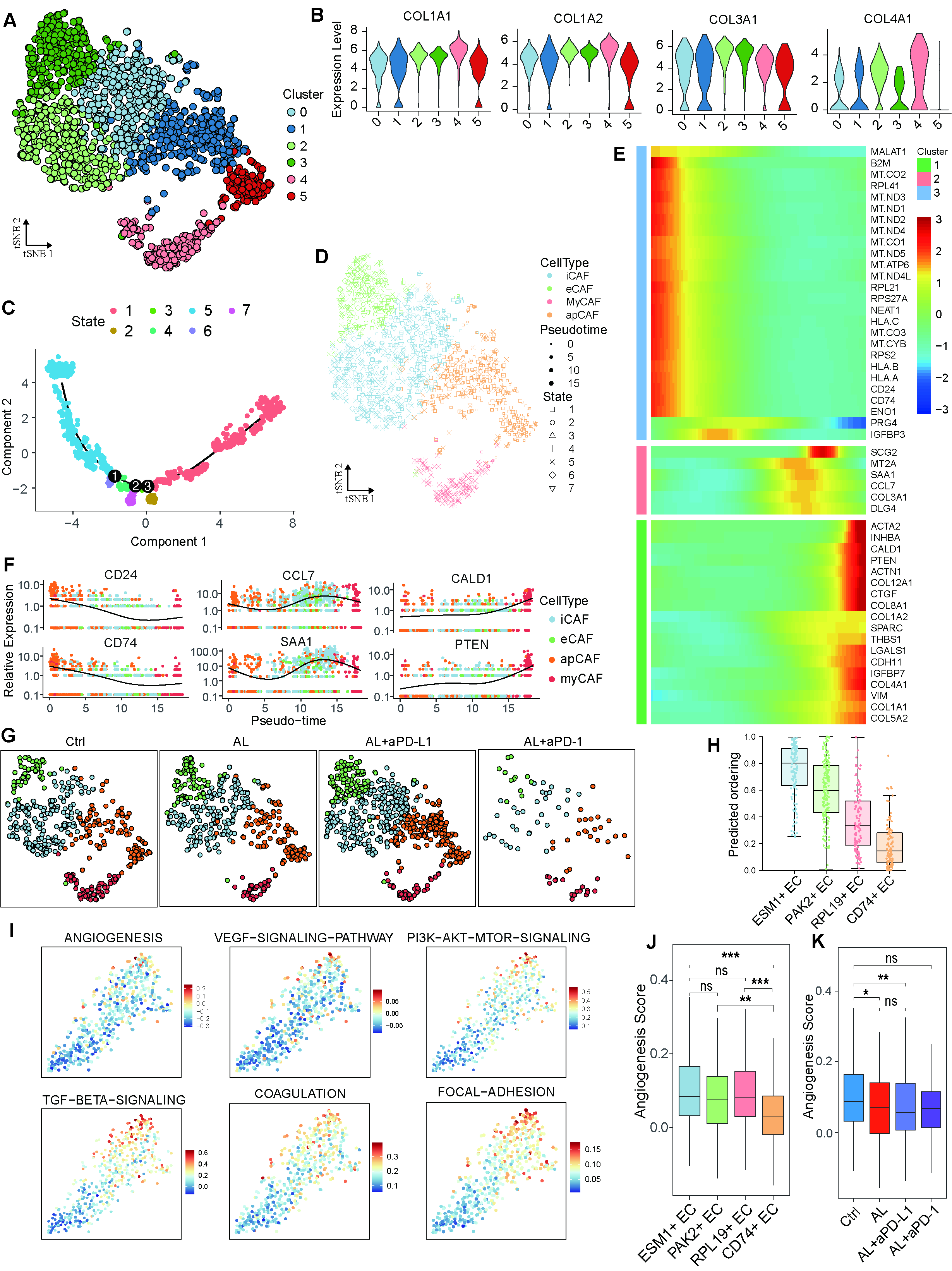


**Figure S4.** **Heterogeneity and Variation of Stromal Cells in the HGSOC TME**

(A) t-SNE plot visualizing fibroblast clusters. (B) Violin plots showing the expression levels of fibroblast marker genes across different clusters. (C) State dynamics along the pseudo-time trajectory in fibroblasts. (D) t-SNE plot showing the differential branches associated with each fibroblast subtype. (E) Hierarchical clustering heatmap showing three subclusters of the top 50 genes along the pseudo-time trajectory of fibroblasts. (F) Expression of key genes involved in fibroblast differentiation. (G) Variation in fibroblast number across different groups. (H) Differential levels of endothelial cell subtypes. (I) Enrichment of angiogenesis-related pathways in endothelial cells. (J, K) Comparison of angiogenesis capability across endothelial cell subtypes and groups.


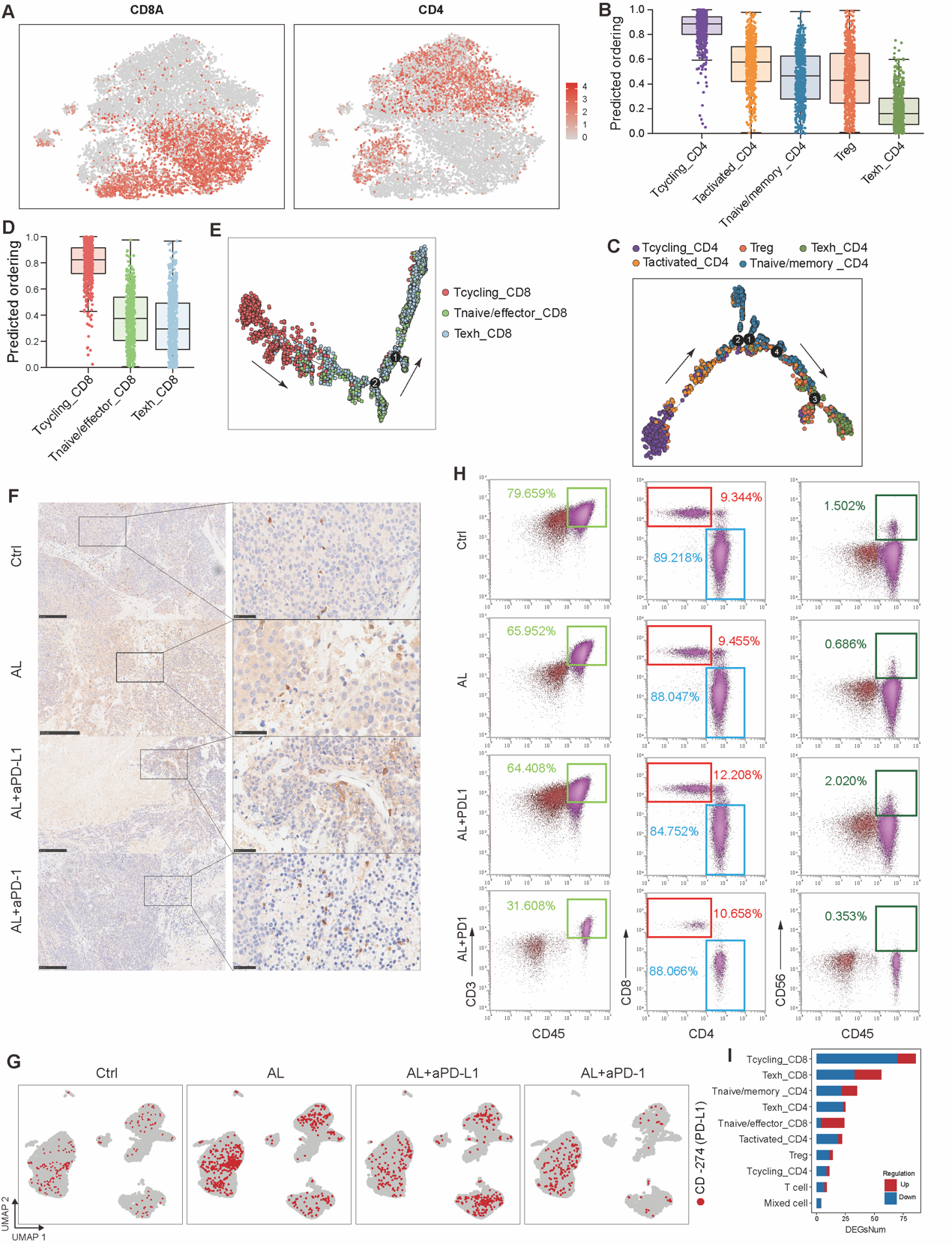


**Figure S5. Variation of T Lymphocytes After Different Treatments in the HGSOC TME**

(A) t-SNE plot showing the expression of CD8A and CD4 in T lymphocytes. (B, C) Pseudo-time trajectory analysis of CD4 T cell subtypes. (D, E) Pseudo-time trajectory analysis of CD8 T cell subtypes. (F) Immunohistochemistry (IHC) showing the expression of PD-L1 across different groups. (G) UMAP visualization showing the expression of CD274 (PD-L1) across different groups. (H) FACS analysis showing the proportions of CD45+CD3+ cells, CD8 cells, CD4 cells, and CD45+CD56+ cells in peripheral blood (PB) across different groups at day 42. (I) Transcriptional changes of T cell subtypes after anlotinib+aPD-L1 treatment. Up indicates up regulated gene number, down indicates down regulated gene number.


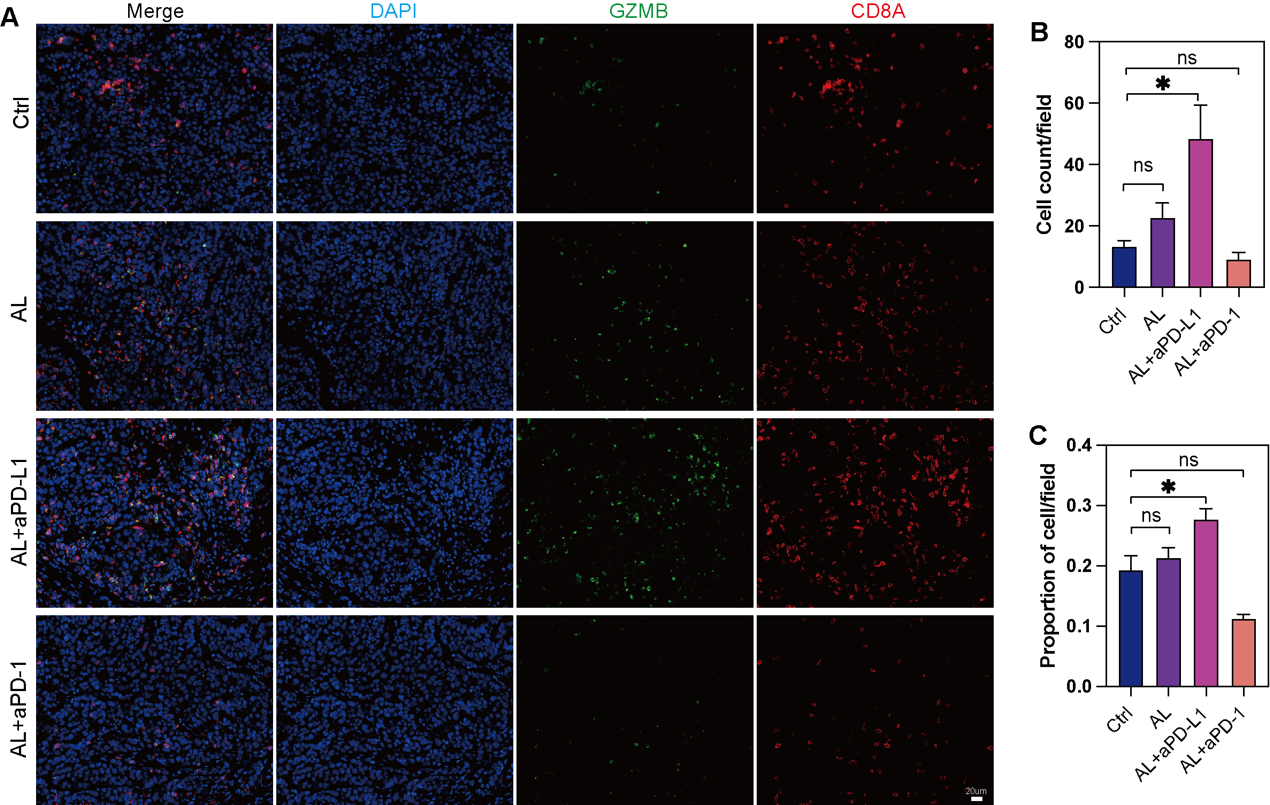


**Figure S6. Variation of CD8+GZMB+ T Lymphocytes After Different Treatments in the HGSOC TME**

(A) Immunofluorescence (IF) images showing CD8⁺GZMB⁺ T cells in different treatment groups. (B) Quantification of CD8⁺GZMB⁺ T cell counts across treatment groups. (C) Proportion of GZMB⁺CD8⁺ T cells relative to total CD8⁺ T cells in each treatment group. (Calculated based on five randomly selected fields per sample, with CD8⁺ T cells and CD8⁺GZMB⁺ T cells counted in each field.)

**
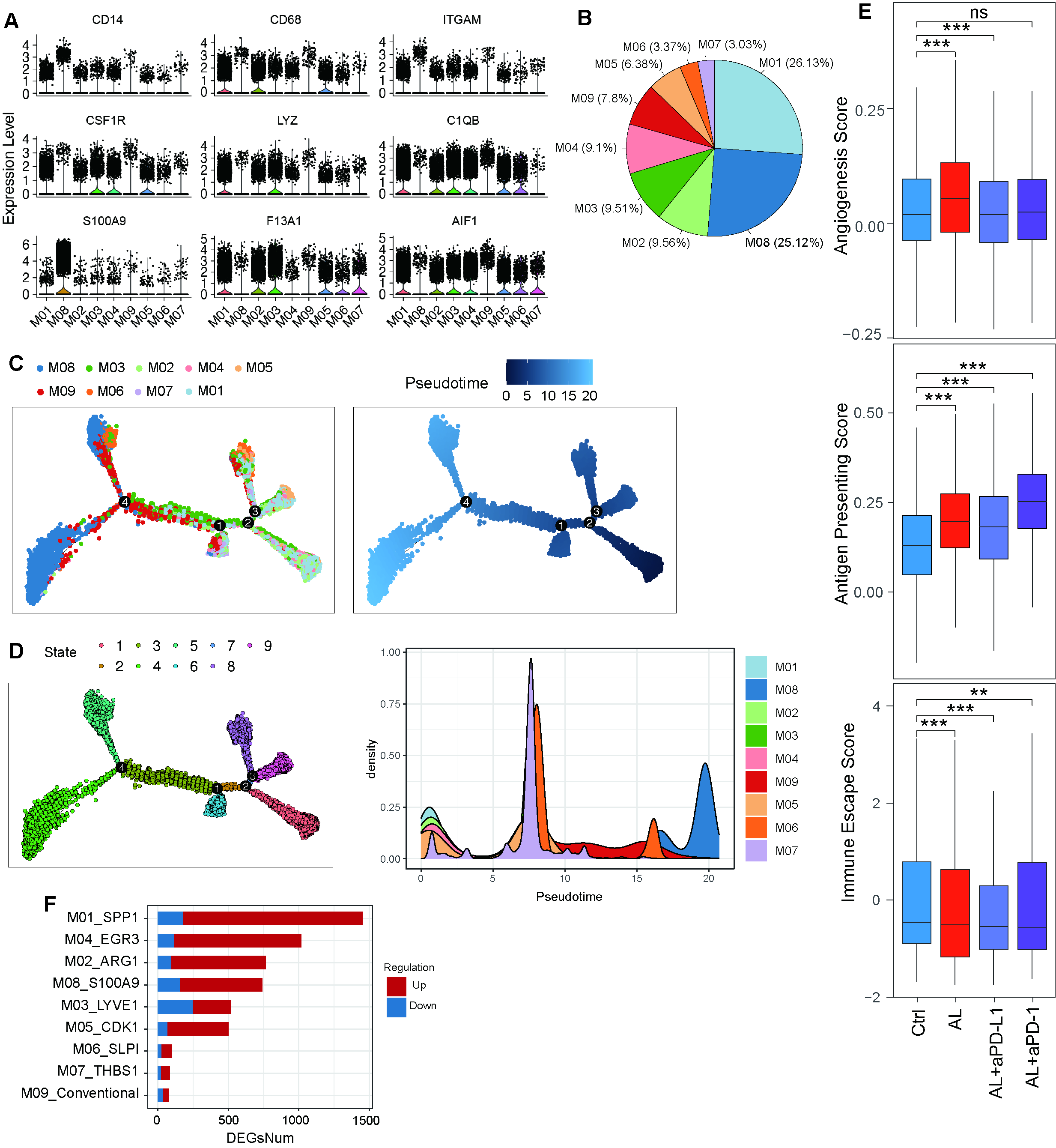
**

**Figure S7. Variation of Myeloid Cells After Different Treatments in the HGSOC TME**

(A) Violin plots showing the expression levels of myeloid marker genes in different myeloid cell subtypes. (B) Pie plot showing the proportion of myeloid cell types. (C) Pseudo-time trajectory analysis of myeloid cell subtypes. (D) State dynamics along the pseudo-time trajectory and differential branches associated with each myeloid cell subtype. (E) Comparison of angiogenesis scores, antigen-presenting scores, and immune escape scores of myeloid cells across different groups. (F) Transcriptional changes of myeloid cell subtypes after anlotinib+aPD-L1 treatment. Up indicates up regulated gene number, down indicates down regulated gene number.

**
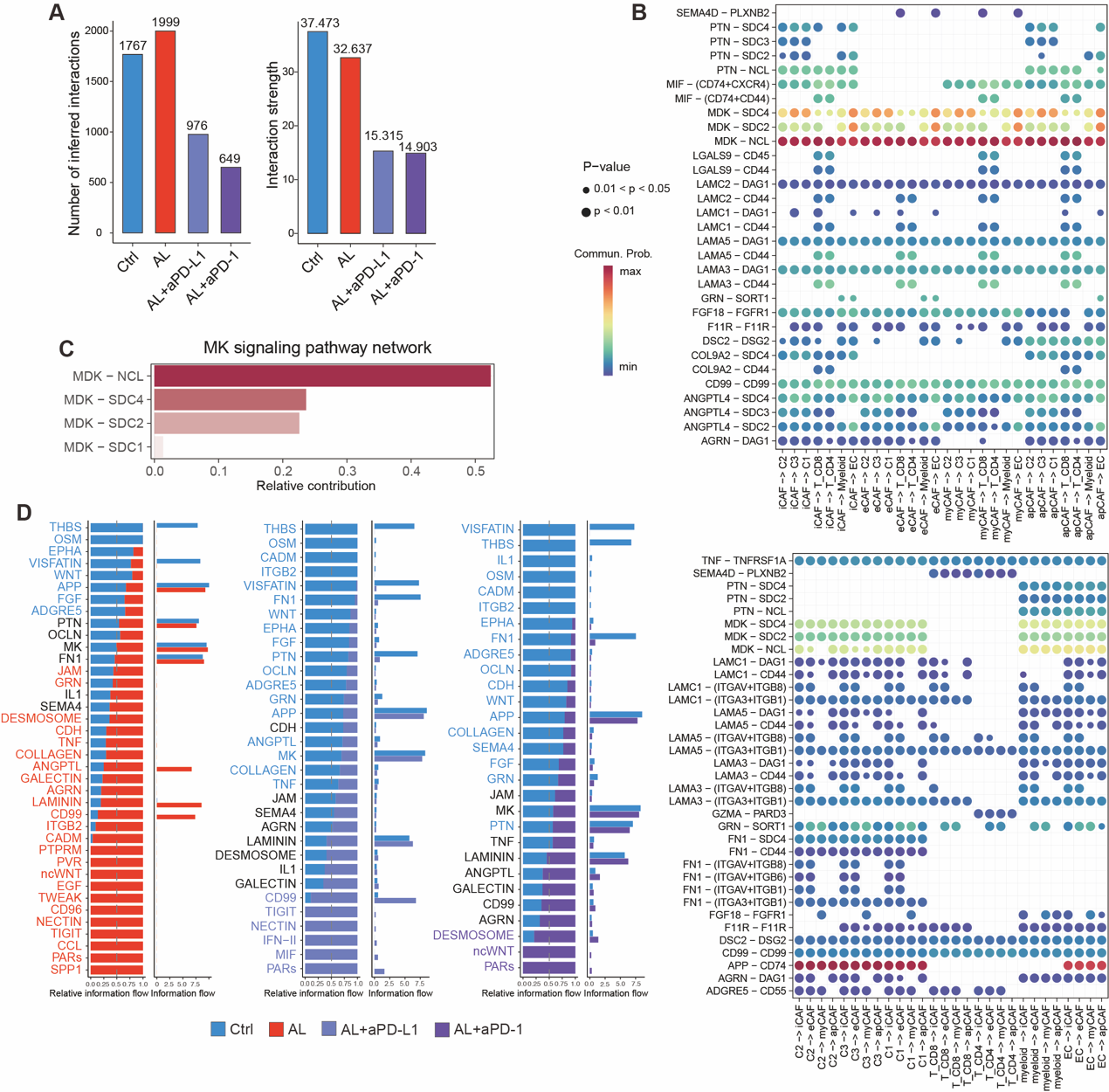
**

**Figure S8. Cell-Cell Communication in the HGSOC TME**

(A) Bar plot showing the number and strength of cell-cell interactions across different groups. (B) Dot plot showing the most significant ligand-receptor pairs between fibroblasts and other cell types. (C) Overview of ligand-receptor pairs involved in MK signaling. (D) Bar plots ranking signaling axes based on overall information flow differences in interaction networks between control, anlotinib, anlotinib + aPD-L1, and anlotinib + aPD-1 groups.


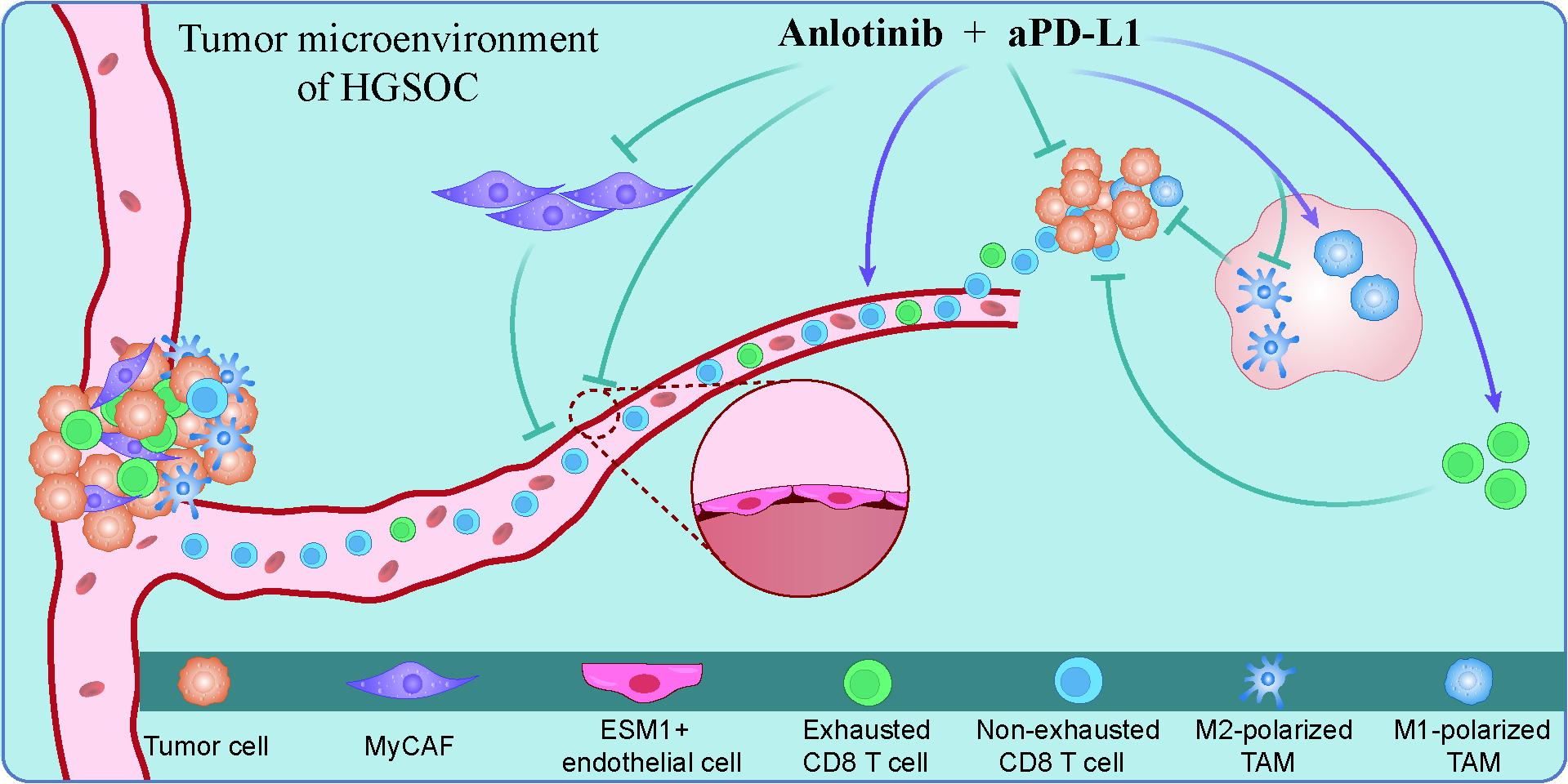


**Figure S9. Visual summary of scRNA-seq findings following anlotinib + aPD-L1 treatment**

**Table S1. Treatment regimen**

| **Group** | **Drug** | **N** | **Drug Dose** | **Dosage regimen** | **Route of administration** |
| --- | --- | --- | --- | --- | --- |
| 1 | control | 5 | -- | -- | -- |
| 2 | Anlotinib | 5 | 0.5mg/kg | QD | p.o. |
| 3 | aPD-L1 + Anlotinib | 5 | 10mg/kg + 0.5mg/kg | BIW + QD | i.v. + p.o. |
| 4 | aPD-1 + Anlotinib | 5 | 10mg/kg + 0.5mg/kg | BIW + QD | i.v. + p.o. |

N, number of PDX models used; QD, once daily; BIW, twice weekly; p.o., oral gavage administration; i.v., intravenous injection.
